# Shear‐Induced Emergence of Aromatic Superlow‐Friction Interfaces in Amorphous Carbon: Triggering Chemical Impurities and Atomic‐Scale Mechanisms

**DOI:** 10.1002/advs.75566

**Published:** 2026-05-25

**Authors:** Takuya Kuwahara, Koki Horiguchi, Leonhard Mayrhofer, Gianpietro Moras, Michael Moseler

**Affiliations:** ^1^ Department of Mechanical Engineering Osaka Metropolitan University Sakai Japan; ^2^ Fraunhofer IWM MikroTribologie Centrum μTC Freiburg Germany; ^3^ Institute of Physics University of Freiburg Freiburg Germany

**Keywords:** diamond‐like carbon, graphene, mechanochemical transformations, quantum chemistry, superlubricity

## Abstract

Incommensurate graphitic interfaces represent an archetypical tribological system to achieve superlow friction. They are currently realized by molecular deposition or lubrication‐triggered, in situ formation of graphitic nanolayers. An alternative route exploits shear‐induced aromatization of amorphous carbon (a‐C). This process occurs directly at surface asperity contacts, relying on chemical impurities like hydrogen and oxygen. Despite the potential of this approach, the lack of mechanistic knowledge, particularly on the role of impurities, is an obstacle to its development and application. To address these gaps, we undertake a systematic simulation study consisting of 1,000 1‐ns‐long quantum‐mechanical molecular dynamics trajectories of sheared a‐C with and without impurities. No aromatic interfaces emerge for four‐valent systems like pure or silicon‐doped a‐C. However, shear‐induced aromatization is consistently observed for impurities with valency lower than four. These cause the formation of voids surrounded preferentially by *sp* ^2^‐hybridized carbon atoms. Upon plastic flow, they stabilize long‐living cavities that evolve into passivated interfaces, rich in polycyclic aromatic structures. The process is driven by shear localization, while passivation by low‐valent impurities prevents reformation of *sp* ^3^‐hybridized domains. This study provides the first comprehensive screening of chemical elements triggering shear‐induced carbon aromatization and is a step forward toward the design of self‐forming and self‐healing, superlubric carbon interfaces.

## Graphical abstract

High‐throughput quantum‐mechanical simulations reveal that amorphous carbon undergoes shear‐driven structural transformation into aromatic, graphene‐like interfaces. This mechanochemical process is governed by dopant chemistry: dopants with valency less than four promote the emergence of superlow‐friction amorphous graphene, whereas tetra‐valent systems, such as pure and silicon‐doped amorphous carbon, suppress it. These results open a mechanochemical route for synthesizing graphene architectures.

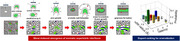

## 1 Introduction

Graphite and graphene are *sp* ^2^‐hybridized, π‐conjugated aromatic carbon allotropes. They represent the most thermodynamically stable carbon phases under ambient conditions, but can transition to *sp* ^3^‐hybridized diamond under high pressure [1, 2]. At the nanoscale, these 2D carbon materials exhibit nearly frictionless sliding (with friction coefficients *µ* below 10^−2^) when two surfaces are in incommensurate contact [3, 4]. This phenomenon arises from strong intralayer covalent bonds, π‐electron delocalization (i.e., a high degree of aromaticity), and weak van der Waals (vdW) interlayer interactions. However, achieving superlubricity of these 2D carbon materials at the macroscale is often hindered by intrinsic surface roughness, defects, and contaminants [5], as well as high normal loads that induce plastic deformation [6]. Thus, developing strategies to preserve or regenerate aromatic interfaces under practical conditions remains a formidable challenge, with significant implications for robust macroscale superlubricity. Current strategies for developing such aromatic interfaces in laboratory‐scale experiments primarily rely on vapor deposition of graphite [7] and polycrystalline graphene [8, 9], and also utilize in situ formation of graphitic layers using aromatic lubricant additives [10] or catalytic conversion of methane gas [11].

An alternative, in situ approach exploits mechanochemical reactions of organic friction modifiers and plastic deformation of amorphous carbon (a‐C) [12, 13]. a‐C is another class of carbon materials, consisting of *sp* ^2^‐hybridized, graphitic, and *sp* ^3^‐hybridized, diamond‐like, carbon atoms [14]. Attaining ultra‐ and super‐low friction requires the absence of chemical bonds across the a‐C sliding interface and an ultrasmooth contacting potential energy corrugation [15]. These prerequisites are typically achieved through passivation of surface dangling bonds by hydrogen atoms and hydroxyl (OH) groups [16, 17]. In contrast, the in situ aromatization process leverages shear‐induced transformation of the a‐C matrix into a graphitic phase [18, 19] and self‐passivation through surface aromaticity, without relying on H/OH passivation. This transformation occurs at asperity‐asperity contacts [19], where high stresses induce bond‐breaking and rehybridization. However, despite the promising potential of shear‐induced aromatization in engineering applications, its underlying atomic‐scale mechanisms remain obscure, posing a critical barrier to the strategic design of robust superlubric interfaces.

A potentially crucial yet overlooked factor in the aromatization process is the role of chemical impurities. While impurities are typically associated with suppression of aromaticity and frictional instability, recent studies suggested that, unexpectedly, chemical impurities can catalyze the formation of aromatic lamella structures [12, 20, 21]. These impurities, such as hydrogen and oxygen [20], can be introduced intentionally during film deposition and also inadvertently supplied by environmental species or lubricants via tribochemical reactions. For example, oxygen can be supplied to the a‐C matrix by environmental O_2_ and H_2_O molecules [16, 22], or the tribochemical decomposition of oxygen‐containing lubricants [12]. Experimental studies have examined the influence of intrinsic and extrinsic “doping” in a‐C structural reorganization [20, 21, 23, 24]. In this article, we use the term dopant to denote chemical impurities introduced both intentionally during deposition and inadvertently through tribochemical processes.

Sliding tests of self‐mated a‐C:H in dry N_2_ showed shear‐induced ordering of *sp* ^2^‐rich graphitic nanolayers in the near‐surface region [19]. Similarly, variations in oxygen and silicon concentrations significantly affect tribochemical reactivities and antifriction properties of a‐C:H interfaces [20], underscoring the importance of the doping element and concentration for sliding interfaces after running‐in. Quantum‐mechanical (QM) molecular dynamics (MD) simulations [12] of a‐C lubricated with glycerol further demonstrated that tribochemical doping with hydrogen and oxygen from glycerol, followed by mechanical mixing, triggers shear‐induced aromatization, resulting in a marked reduction in friction. Nevertheless, the mechanisms by which dopants catalyze the formation of superlubric aromatic interfaces upon shear plastic deformation of a‐C remain unknown.

To solve this puzzle, we perform a systematic tight‐binding QM MD simulation study of a‐C with and without dopants under shear deformation. Comprehensive analyses of one thousand 1‐ns‐long QM MD trajectories for eight doping elements, supplemented by first‐principles density functional theory (DFT) MD simulations, reveal the pivotal role of dopants with valency less than four (e.g., H and O) in the shear‐induced emergence of aromatic interfaces. In contrast, no such interfaces form in tetravalent systems like pure or Si‐doped a‐C. The lower‐valent dopants terminate carbon dangling bonds, stabilizing *sp* ^3^‐C at their bonding sites while preferentially inducing *sp* ^2^‐C in adjacent regions. Under plastic shear flow, these lower‐valent dopants facilitate the nucleation of nano‐voids and their stabilization and growth, leading to the emergence of aromatic rings and superlubric amorphous graphene‐like interfaces [25, 26]. Our results provide a first systematic virtual screening of chemical elements that can either trigger or hinder shear‐induced aromatization and offer a mechanochemical pathway for synthesizing nanographene and other 2D nanomaterials under mechanical stress. This study unveils an important ingredient to an atomistic recipe for designing shear‐driven nanostructures in a‐C through controlled intrinsic and extrinsic doping, potentially extending to more complex ternary systems.

## 2 Results

**FIGURE 1:**
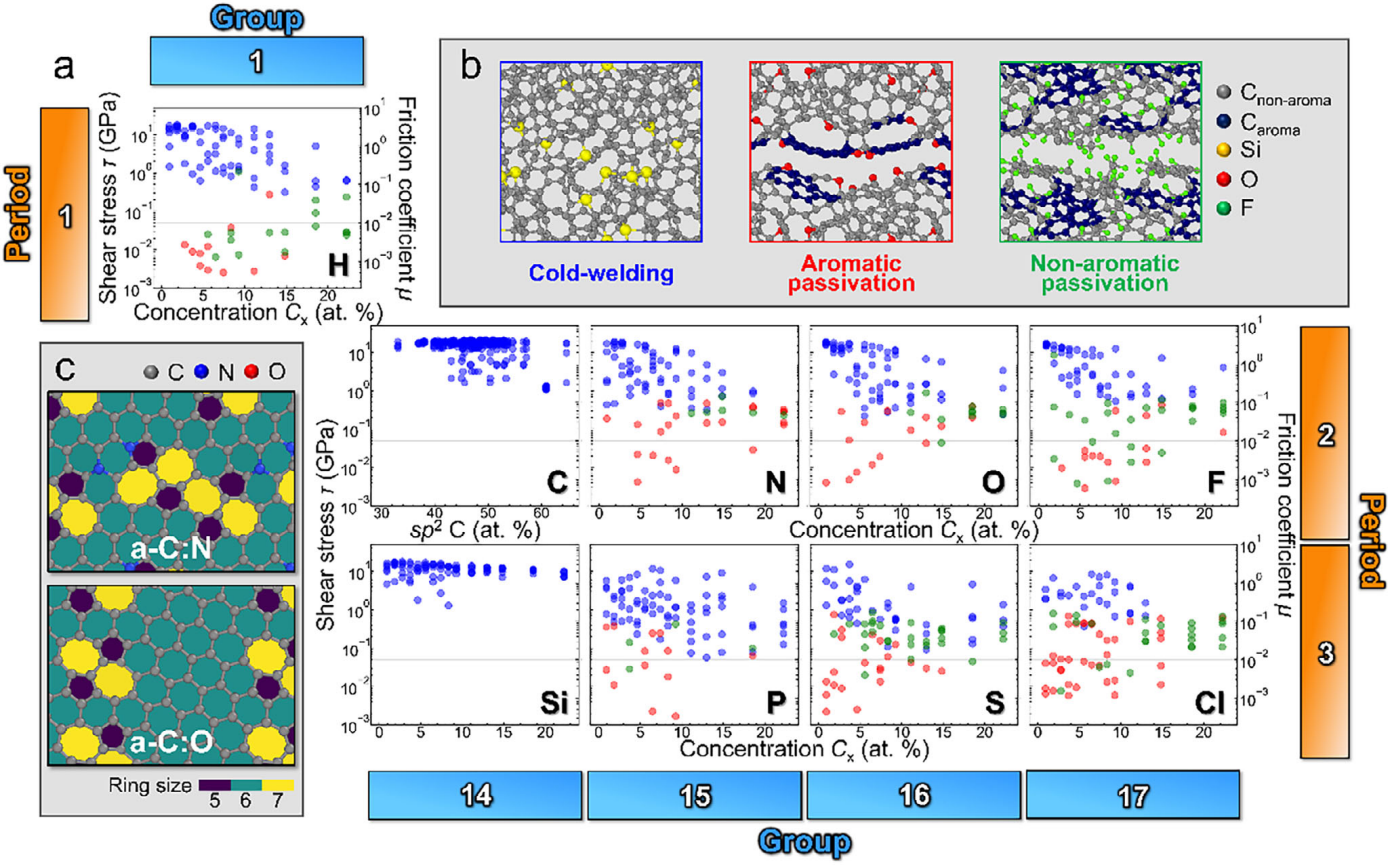
(a) Structure‐property maps relating shear stress (friction) and doping concentration arranged as a periodic table of dopants. Each marker represents the result of an independent 1‐ns‐long DFTB MD trajectory and is color‐coded according to the structural analysis described in Ref. [29]: CW (blue), aromatic passivation (red), and non‐aromatic passivation (green). For each dopant concentration, 6 MD trajectories are generated with different initial configurations. Shear stresses τ and friction coefficients μ (= τ/*P_N_* ) are plotted on the left and right y‐axes, respectively. The horizontal lines correspond to the superlubricity limit μ  =  0.01. The degree of aromaticity is determined using a geometrical descriptor [30] (more details in Section 4). (b) Representative atomic configurations of CW for a‐C:Si with *C*_Si_ =  4.6 at. %, aromatic passivation for a‐C:O with *C*_O_ =  6.5 at. %, and non‐aromatic passivation interfaces for a‐C:F with *C*_F_ =  22.2 at. %. (c) Representative atomic configurations from aromatic passivation trajectories for a‐C:N (top) and a‐C:O (bottom) with *C*_X_ =  4.6 at. %.

To elucidate the mechanistic roles of dopant elements in shear‐induced aromatization, we perform 1,000 1‐ns‐long QM MD simulations using the density‐functional‐based tight‐binding (DFTB) method [27]. The simulations encompass eight dopant elements embedded in a representative volume of a‐C that is subjected to homogeneous shear flow [28]. The DFTB results are validated by ab initio DFT MD (Figure S1). Our simulations reveal a pronounced influence of the dopants and of their concentrations on shear‐induced aromatization. An overview of all the MD simulations is provided in Figure 1, which displays structure‐property maps relating shear stress and dopant concentration for the dopants considered here—H, N, O, F, Si, P, S, and Cl. In addition, the shear stress of undoped a‐C as a function of sp^2^‐hybridized carbon content is shown in Figure 1. The structure‐property maps are organized according to the position of the dopant element in the periodic table. Hereafter, a‐C doped with the element X and its concentration is denoted as a‐C:X and *C*_X_, respectively. In these maps, each marker represents the shear stress τ and friction coefficient μ calculated from the final 0.1 ns of each individual MD trajectory, with colors indicating the friction regime. Here, μ is defined as τ/*P_N_*, where *P_N_* is the applied normal pressure. Friction regimes are classified into cold‐welding (CW), aromatic passivation, and non‐aromatic passivation regimes, based on structural analyses of the sliding interfaces (see Section 4 and Ref. [29] for further details). The CW regime is characterized by high friction, whereas the aromatic and non‐aromatic passivation regimes represent low‐friction interfaces. In the non‐aromatic passivation regime, the surface termination is dominated by functional groups such as C─H in a‐C:H or C═O in a‐C:O [22]. In contrast, the aromatic passivation regime is characterized by low‐friction interfaces between polycyclic aromatic surfaces. The stresses in these structure‐property maps show large scatter, which arises from the stochastic nature of the aromatization process and the resulting atomic structures of the interface. The nucleation and growth of an aromatic interface require mechanical mixing of the CW system, which is inherently random, and the large scatter reflects the survival probability of the CW state. The resulting atomic structures vary in ring statistics, degree of aromaticity, and atomic‐scale roughness [29] and thus lead to different frictional responses.

The first important observation is that systems composed of tetravalent elements only do not exhibit the formation of low‐friction passivated interfaces within the simulation timescale: the shear stress τ remains unaffected by the Si concentration *C*_Si_ in a‐C:Si and by the *sp* ^2^ C content in undoped a‐C. In contrast, dopants with valency less than four promote shear‐induced formation of low‐friction interfaces due to both non‐aromatic and aromatic passivation. For these low‐valent dopants, τ in the CW regime and the number of trajectories that remain in the CW regime throughout the simulation decrease sharply with increasing *C*_X_. Moreover, for mono‐ (H, F, and Cl) and di‐valent dopants (O and S), the aromatic passivation is observed at lower *C*_X_ (≲15 at. %), while the non‐aromatic passivation is prominent at higher *C*_X_ (≳15 at. %), indicating the existence of optimal concentrations of these mono‐ and di‐valent dopants for facilitating the formation of polycyclic aromatic interfaces.

a‐C samples doped with trivalent elements, such as N and P, are characterized by a distinct behavior. The number of non‐aromatic passivation trajectories (green markers) is significantly lower than in the mono‐ and di‐valent element cases. Particularly for trivalent N, the aromatic‐passivation regime extends to *C*_X_ > 20 at. %. This can most likely be attributed to the ability of N to participate in aromatic rings. For instance, N can form polycyclic graphitic C_x_N_y_ structures, whereas O is less likely to participate in aromatic rings, as illustrated by representative atomic configurations in Figure 1c. These surfaces consist of 5‐ to 7‐membered rings, resembling amorphous graphene [26].

**FIGURE 2:**
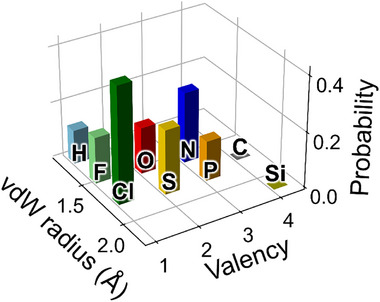
Probability of aromatic passivation as a function of valency and vdW radius.

The role of dopants as aromatization promoters or inhibitors is further analyzed by calculating the aromatization probability *P*_a_, defined as the ratio of aromatization trajectories to the total number of trajectories for each dopant (Figure 2). The results indicate that chemical characteristics of dopants, e.g., valency and vdW radius, influence the emergence of polycyclic aromatic interfaces. For mono‐ and di‐valent dopants, *P*_a_ increases with vdW radius. In contrast, trivalent dopants exhibit the opposite trend. Within the same period, *P*_a_ generally increases with decreasing valency, except for N, whose strong tendency to form aromatic rings facilitates shear‐induced aromatization. As already mentioned above, tetravalent a‐C and a‐C:Si systems exhibit *P*_a_ =  0.

**FIGURE 3:**
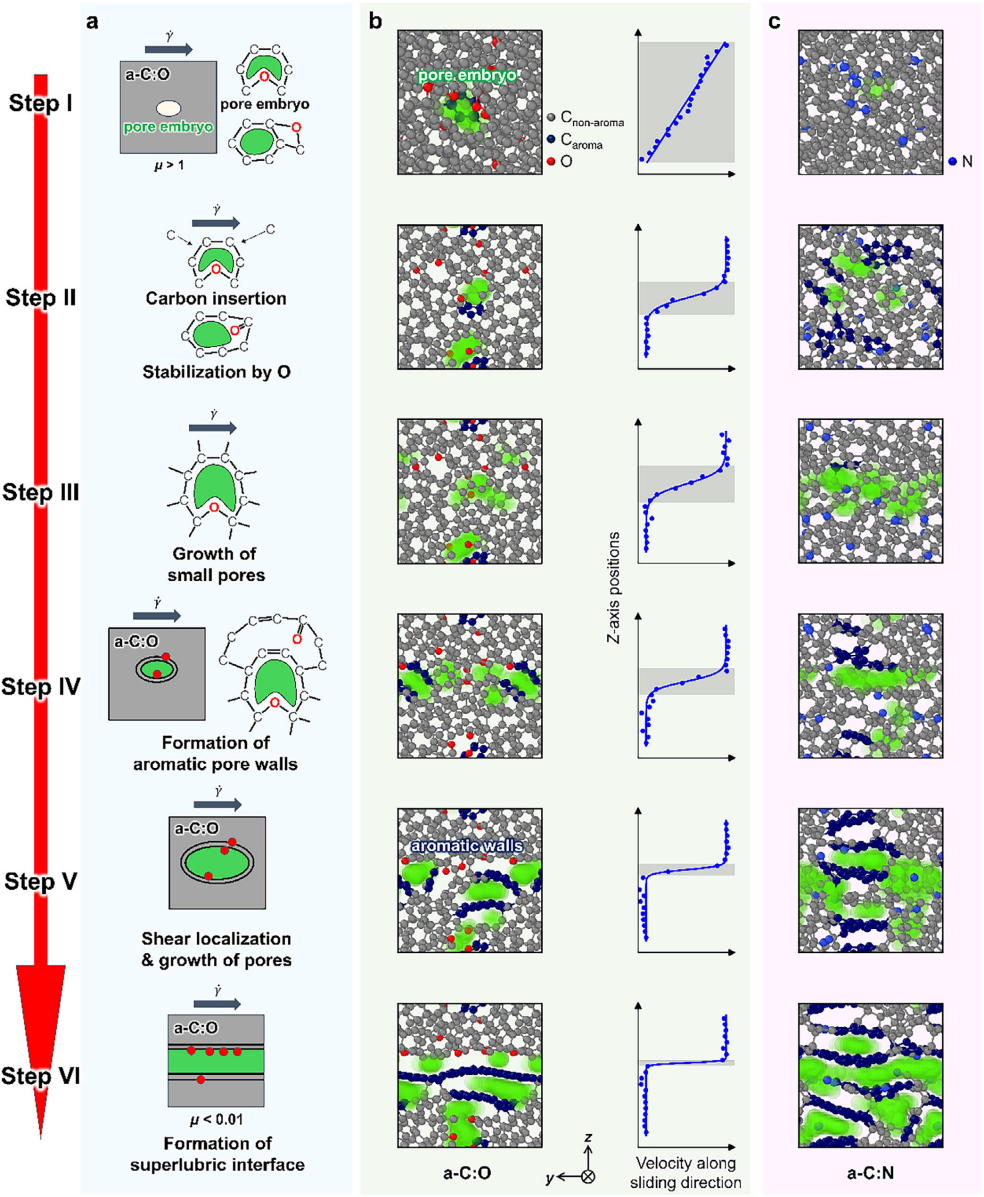
(a) Conceptual six‐step model and (b) representative snapshots of shear‐induced aromatization and velocity profiles along the sliding direction in a‐C:O. In panel b, the snapshots are extracted from an MD trajectory of a‐C:O with *C*_O_ =  4.6 at. %. For visualization of pores, individual pore elements are represented by semi‐transparent green spheres with a radius of 1 Å. Non‐aromatic and aromatic C atoms are represented by grey and navy‐blue spheres, respectively. Geometrical criteria to evaluate the degree of aromaticity for each C atom are described in Section 4. Oxygen atoms are represented by red spheres. The simulation cell is replicated once in all directions. A homogeneous shear flow is generated along the *x* direction with Lees‐Edwards boundary conditions (BCs) [28]. In the velocity profiles, grey rectangles represent the shear accommodation regions. (c) Representative snapshots of shear‐induced aromatization in a‐C:N with *C*_N_ =  4.6 at. %.

Comprehensive analyses of our 1,000 QM MD trajectories unveil a generalized mechanistic framework of how the synergy between dopants and plastic deformation promotes or hinders shear‐induced aromatization. Figure 3 provides a six‐step conceptual model of this process (Figure 3a), accompanied by key snapshots extracted from a representative QM MD trajectory of a‐C:O with *C*_O_ =  4.6 at. % (Figure 3b). We note that this conceptual model applies to all dopants with valency smaller than four analyzed in this work (see, for instance, Figure 3c for N). Snapshots for the elements H, F, P, S, and Cl are shown in the Supporting Information.

Step I: The thermodynamically metastable a‐C bulk inherently contains nano‐voids, which we call “pore embryos”, where *sp* ^2^ aromatic C and O atoms (or N) are often localized, as depicted in the first row of Figure 3.
Step II: Plastic shear deformation induces *sp* ^3^‐to‐*sp* ^2^ rehybridization [31], resulting in a local density decrease. Pore embryos and aromatic rings transiently form but rapidly collapse under plastic shear flow.
Step III: Pore embryos grow gradually as O atoms become part of pore walls, accompanied by C─C bond scission (see the second and third panels of Figure 3a,b). For instance, the cleavage of C─C bonds between *sp* ^3^ C atoms creates *sp* ^2^ C atoms in the pore wall. O insertion passivates a dangling bond, forming C─O─C or C═O groups while leaving adjacent *sp* ^2^ C atoms unpassivated. This local O passivation creates excluded volumes. In the case of nitrogen doping, NC_3_ groups form (Figure 3c).
Step IV: Continuous shear promotes further O (or N) insertion and conversion of non‐aromatic carbon in pore walls into aromatic ones (see the fourth row of Figure 3).
Step V: The O (or N) accumulation and formation of aromatic rings induce shear localization, facilitating further pore growth (fifth row of Figure 3).
Step VI: In the final stage, the few remaining weak interlayer C─C bonds are cleaved, converting non‐aromatic into aromatic rings and leading to the emergence of an “amorphous graphene” surface. The underlying layer is passivated with O (or N) functional groups, preventing reversion to a *sp* ^3^‐hybridized state under high contact pressures. The topmost layer is partially aromatic and connected to the second aromatic layer through *sp* ^3^‐hybridized domains, reminiscent of diamond‐to‐graphite transitions [32].

**FIGURE 4:**
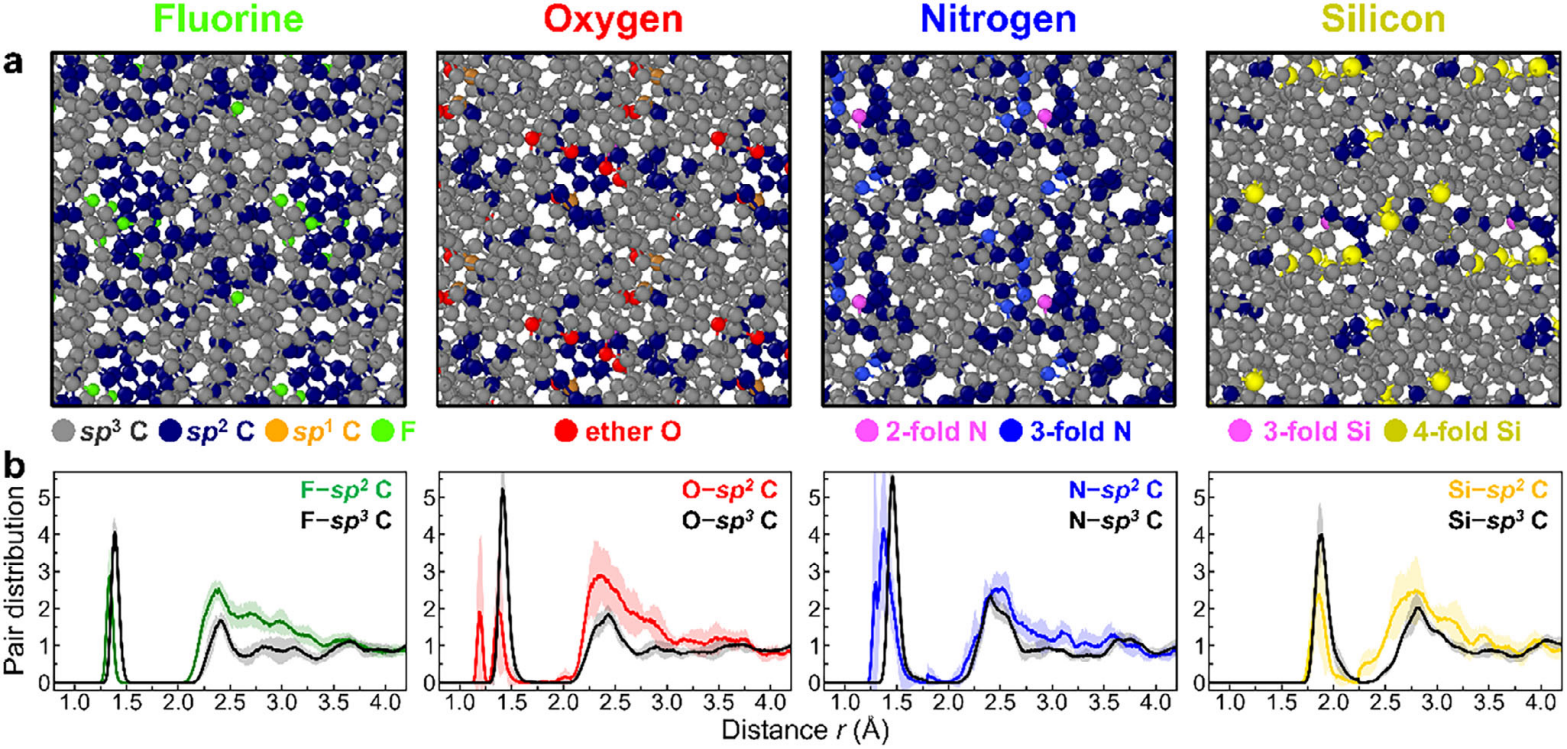
Chemical structures (a) and pair correlation functions (b) of a‐C:F, a‐C:O, a‐C:N, and a‐C:Si with *C*_X_ =  4.6 at. % after preparation of the samples via a melt‐quench procedure and subsequent thermal and pressure equilibration at 300 K and 5 GPa.

**FIGURE 5:**
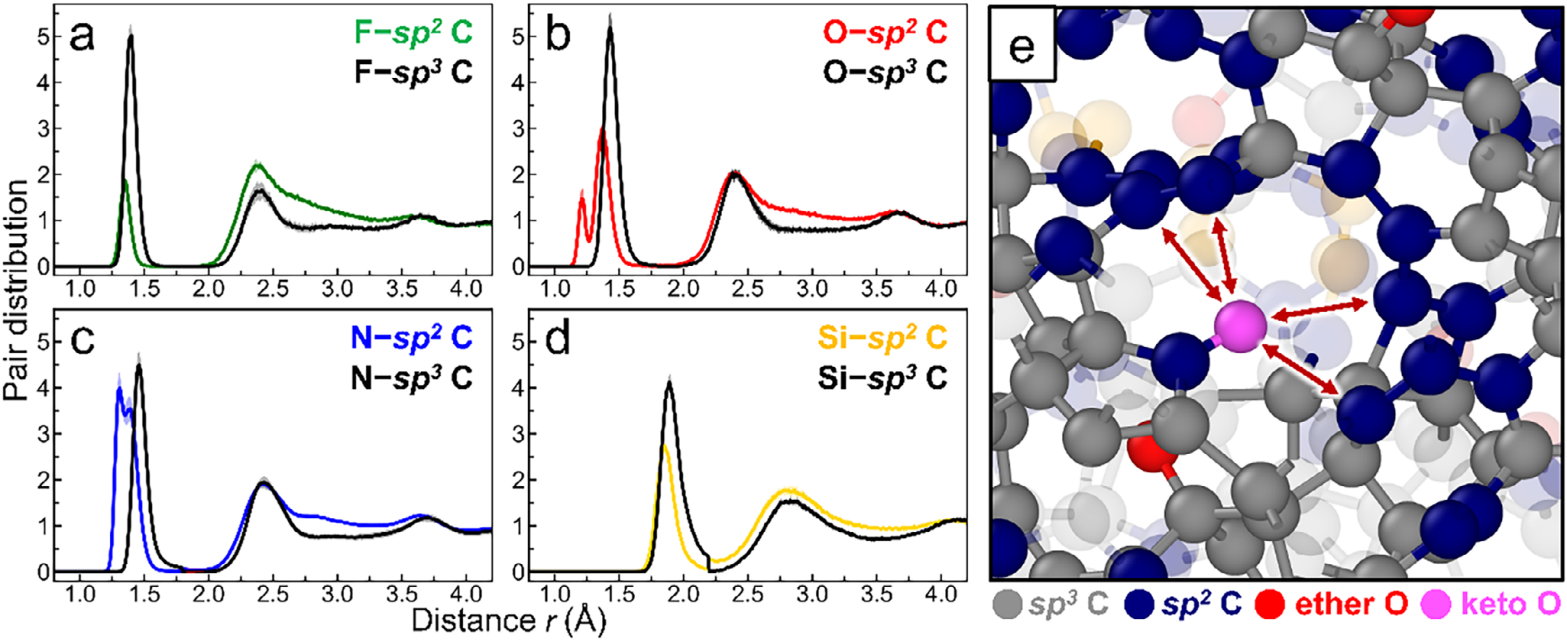
Pair correlation functions between dopants and *sp* ^2^ C, $g_{X-sp^{2}\;C}$, and between dopants and *sp* ^3^ C, $g_{X-sp^{3}\;C}$ for (a) a‐C:F, (b) a‐C:O, (c) a‐C:N, and (d) a‐C:Si at *C*_X_ =  4.6 at. %. These are calculated every 0.2 ps and averaged over the time interval from 200 to 450 ps with a bin width of 0.01 Å. Shaded regions represent standard deviations of six independent MD trajectories. (e) Representative snapshot showing a keto oxygen (magenta) and adjacent *sp* ^2^ C atoms (navy blue). Brown arrows indicate *sp* ^2^ C atoms located at distances of 2.6–2.9 Å from the keto O atom, corresponding to the shoulder observed for $g_{O-sp^{2}\;C}$ in panel b.

**FIGURE 6:**
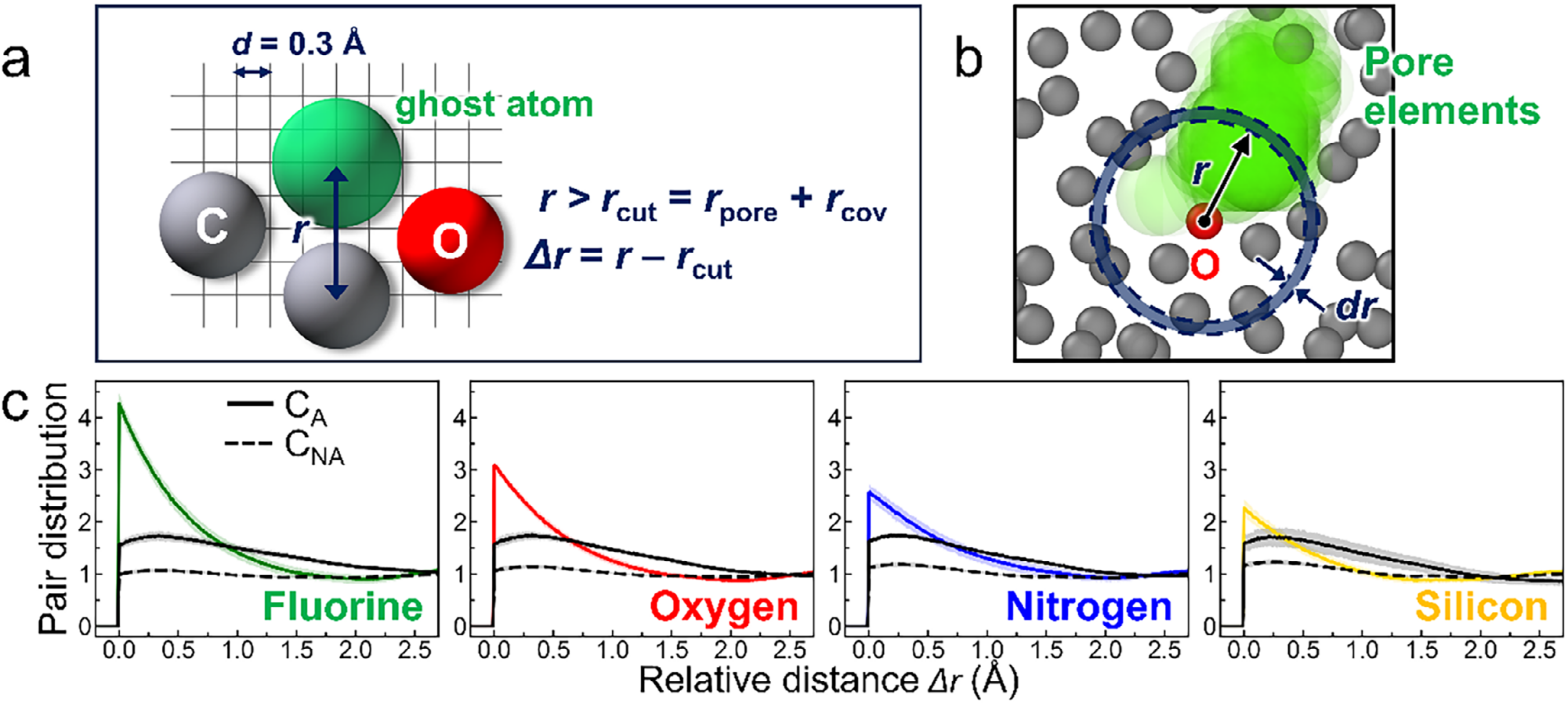
Pore statistics for a‐C:F, a‐C:O, a‐C:N, and a‐C:Si with *C*_X_ =  4.6 at. %. Schematic illustrations of (a) pore and (b) pair correlation function analysis. Details of the pore analysis are provided in the main text and Section 4. (c) Pair correlation functions of dopant‐pore *g*_X − p_(*r*) (colored), aromatic C‐pore $g_{C_{A}-p}\left(r\right)$ (solid black), and non‐aromatic C‐pore $g_{C_{\mathrm{NA}}-p}\left(r\right)$ (dashed). This analysis is carried out every 0.2 ps between 200 and 450 ps. Shaded regions represent standard deviations of six independent MD trajectories. The degree of aromaticity of each atom at time *t* is evaluated using a geometrical descriptor [30].

**FIGURE 7:**
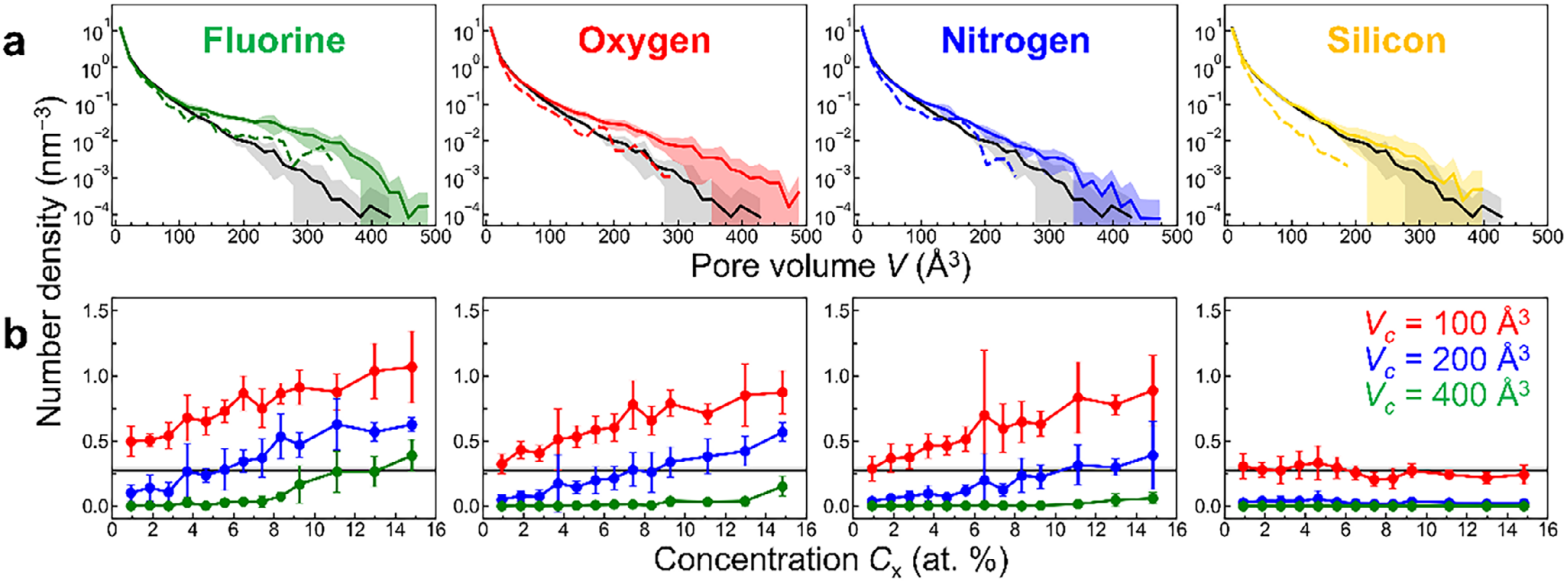
(a) Number densities (nm^−3^) of pores as a function of pore volume *V*_pore_ for F, O, N, and Si with *C*_X_ =  4.6 at. %. Analyses are carried out between 200 and 450 ps every 0.2 ps. Solid lines and shaded regions represent averages and standard deviations of six independent MD trajectories, respectively. The black lines and shaded regions represent the reference data for a‐C. Dashed lines represent averages calculated between 0 and 100 ps. (b) Number densities of pores with $V_{\mathrm{pore}}\geq 100\;{Å}^{3}$ as a function of *C*_X_. Markers and error bars represent averages and standard deviations of six independent MD trajectories, respectively. The black lines and shaded regions represent the reference data for pure a‐C.

**FIGURE 8:**
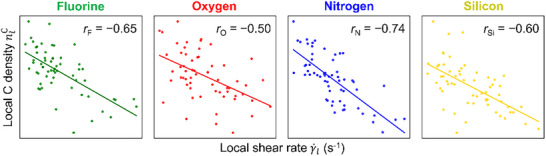
Spatial correlations between local shear rates ${\dot{\gamma }}_{l}$ and local number densities of C atoms $n_{l}^{C}$ and corresponding Pearson correlation coefficients for F, O, N, and Si dopants with *C*_X_ =  4.6 at. %. Solid lines represent the linear fits to the scattered data. Markers denote averages between 200 and 450 ps for each bin along the *z* axis.

**FIGURE 9:**
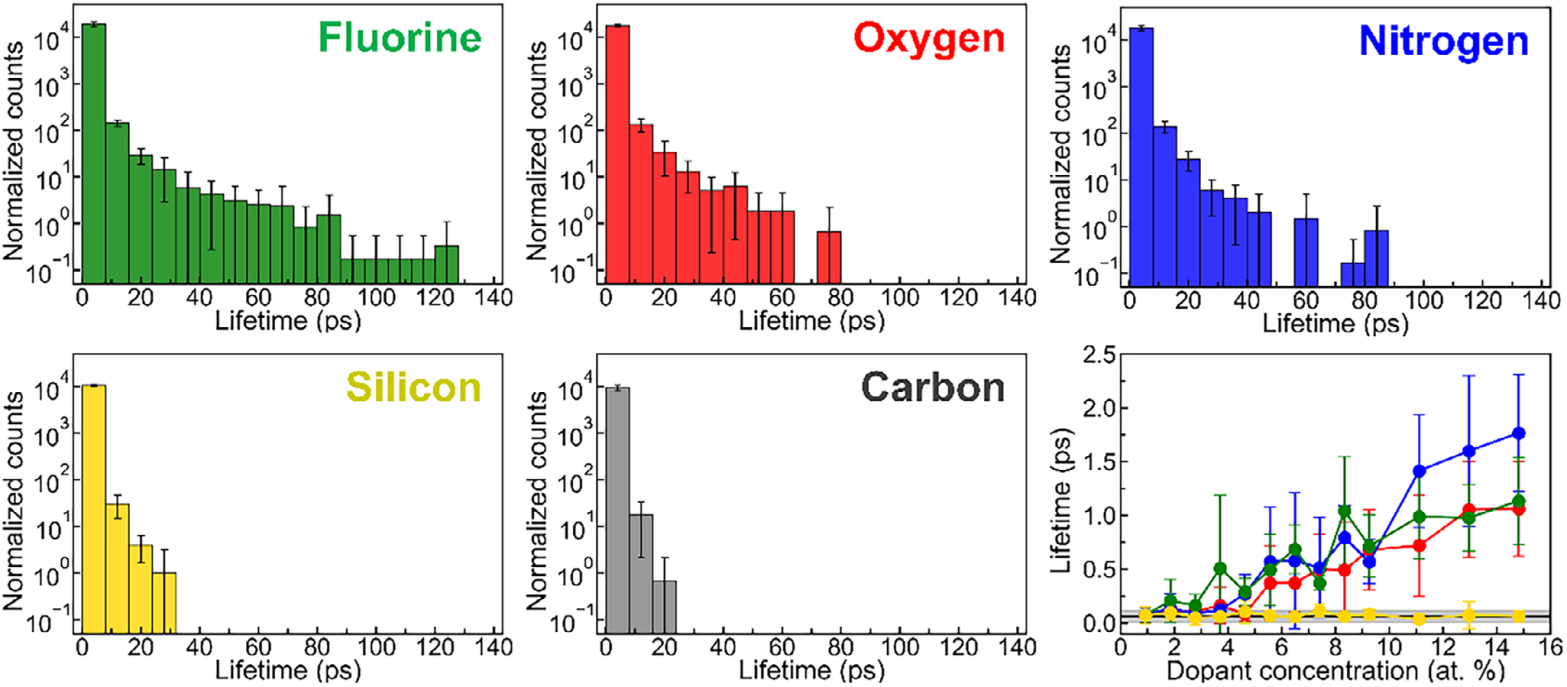
Lifetime distributions τ_*a*_ of aromatic C atoms for F, O, N, and Si dopants with *C*_X_ =  4.6 at. % as well as for pure a‐C, and averaged lifetimes 〈τ_*a*_〉 as functions of *C*_X_. Analyses are conducted for CW systems between 200 and 450 ps every 0.2 ps, i.e., before a low‐friction interface forms. For each bin, the counts are normalized to the total number of Cwa atoms. In the bar plots, values and error bars represent averages and standard deviations over six independent MD trajectories. In the line plot, black horizontal lines and shaded gray regions indicate the averages and standard deviations for pure a‐C, respectively.

The six‐step model was derived by a visual inspection of the numerous trajectories that lead to the formation of aromatic interfaces. This must be corroborated by a systematic statistical analysis of the decisive mechanisms. In the following analyses (Figures 4, 5, 6, 7, 8, 9), we examine how the different dopants influence the individual aromatization steps under seven main aspects:

1. Low valency dopant atoms promote sp^2^‐rich environments during step I, and this introduces pore embryos: this can be proven by considering the spatial correlations of dopant atoms with *sp* ^2^ C and *sp^3^* C (Figures 4 and 5).
2. Low valency dopants stabilize small pores during step II: this should be reflected in the spatial correlations of dopant atoms with void regions (Figure 6).
3. The growth of small pores in step III: this should be underpinned by an analysis of the evolution of average pore size (Figure 7).
4. The aromatization of the pores in step IV: studying the spatial correlation of aromatic C atoms with pores is essential to back up this claim (Figure 6).
5. Low‐valency atoms stabilize neighboring aromatic rings in steps IV and V: this can be verified by considering the lifetime of C atoms in an aromatic state (Figure 9).
6. The growth of large pores in step V: an inspection of the pore size distributions and how they evolve is needed to corroborate this aspect (Figure 7).
7. Large pores lead to shear localization and the formation of aromatic sliding interfaces (step VI): analyzing anti‐correlations between local shear rates and local densities of C atoms and lifetimes of aromatic C atoms can support this assertion (Figure 8).

For clarity and focus, we concentrate on four dopants with different valencies, namely F, O, N, and Si, and on one concentration *C*_X_ =  4.6 at. %. Results for the remaining dopants and concentrations are provided in Figures S2–S5. Figure 4a shows the chemical structures of a‐C:X bulk systems with *C*_X_ =  4.6 at. % prior to plastic shear deformation, highlighting how dopants influence local carbon rehybridization. Although carbon hybridization depends on the quenching rate and density in the preparation process [33], we consider a comparison of systems that were prepared with an identical protocol a useful approach to analyze general chemical trends. Indeed, our a‐C:X models at *C*_X_ =  4.6 at. % exhibit differences in their *sp* ^2^ C contents: a‐C:F 43.7 ± 7.4%, a‐C:O 28.4 ± 2.5%, a‐C:N 30.5 ± 4.3%, and a‐C:Si 31.7 ± 5.2%. The higher *sp* ^2^ content for F is due to its mono‐valency and relatively large atomic size, creating excluded volumes that effectively promote *sp* ^2^ C formation in adjacent regions. The snapshots qualitatively indicate that *sp* ^2^ C atoms are localized around dopants, while *sp* ^3^ C atoms remain concentrated in dopant‐free regions. Dopants seem to be spatially clustered rather than homogeneously distributed. Since *sp* ^2^ C atoms tend to preferentially form near dopants, local dopant accumulation can expand *sp* ^2^ C domains. These observations are confirmed statistically by calculating a pair correlation function $g_{X-sp^{2}\;C}\left(r\right)$ between dopant and *sp* ^2^ C atoms (Figure 4b), which is defined as:

(1) $$g_{X-sp^{2}\;C}\;\left(r\right)=\frac{1}{n_{t}}\;\sum_{t}^{n_{t}}\sum_{i}^{n_{X}}\sum_{j}^{n_{sp^{2}\;C}\left(t\right)}\frac{V\left(t\right)}{n_{X}n_{sp^{2}\;C}\left(t\right)}\frac{\delta_{X-sp^{2}\;C}\left(r,t\right)}{4\pi r^{2}dr},$$

where *n_t_* is the number of trajectory frames, *n*_X_ is the number of dopant atoms, $n_{sp^{2}\;C}\left(t\right)$ is the number of *sp* ^2^ C atoms, and *V*(*t*) is the system volume at time *t*. Similarly, the pair correlation function $g_{X-sp^{3}\;C}\left(r\right)$ between *sp* ^3^ C and dopant X can be calculated. For F, the first peaks in both $g_{F-sp^{2}\;C}$ and $g_{F-sp^{3}\;C}$ correspond to F─C bonds, and the more pronounced peak in $g_{F-sp^{3}\;C}$ suggests a higher likelihood of F binding to *sp* ^3^ C atoms. The second peak in $g_{F-sp^{2}\;C}$ is significantly larger and broader than that in $g_{F-sp^{3}\;C}$, indicating that *sp* ^2^ C atoms form preferentially at second‐nearest‐neighbor positions of dopant atoms. For O, a significant difference between $g_{O-sp^{2}\;C}$ and $g_{O-sp^{3}\;C}$ is observed in the nearest neighbor peaks. The first peak in $g_{O-sp^{3}\;C}$ at r=1.43Å corresponds to ether C─O─C groups, while $g_{O-sp^{2}\;C}$ exhibits peaks at *r*  =  1.23 and 1.36Å, which can be attributed to keto O═C and aromatic O─C bonds in furan, respectively. The comparison of the second peaks between $g_{O-sp^{2}\;C}$ and $g_{O-sp^{3}\;C}$ shows the same trend as that observed for F. For N, the first peaks in $g_{N-sp^{2}\;C}$ lie at *r*  =  1.27 and 1.35Å, at a smaller distance than the first peak in $g_{N-sp^{3}\;C}$ (r=1.48Å), indicating imine N═C and aromatic N─C bonds, respectively, while the latter corresponds to an amine bond. The difference in the second peaks between $g_{N-sp^{2}\;C}$ and $g_{N-sp^{3}\;C}$ is less pronounced for N than for F and O. For Si, the first peaks in both $g_{\mathrm{Si}-sp^{2}\;C}$ and $g_{\mathrm{Si}-sp^{3}\;C}$ correspond to Si─C bonds. The second peaks in both $g_{\mathrm{Si}-sp^{2}\;C}$ and $g_{\mathrm{Si}-sp^{3}\;C}$ exhibit similar intensities, but that in $g_{\mathrm{Si}-sp^{2}\;C}$ is slightly broader than $g_{\mathrm{Si}-sp^{3}\;C}$. These statistical results corroborate the qualitative trends observed in the snapshots shown in Figure 4a.

This structural heterogeneity induced by the presence of dopants can still be detected in the a‐C:X systems under plastic shear deformation. Figure 5a–d present a comparison between $g_{X-sp^{2}\;C}$ and $g_{X-sp^{3}\;C}$ at *C*_X_ =  4.6 at. % for dopants X = F, O, N, Si. The presence of a shoulder between *r*  =  2.5 and 3.5Å (i.e., a visible flattening of the decaying curve), corresponding to the separation between a dopant atom and adjacent *sp* ^2^ C atoms not directly bonded to it, remains a notable feature in $g_{X-sp^{2}\;C}$ for the low‐valent dopants. An example of such a configuration is depicted in Figure 5e, with red arrows indicating *sp* ^2^ C atoms located at 2.6Å≲r≲2.9Å around a keto O atom. In contrast, no shoulder is observed for Si, implying that tetravalent Si atoms hinder the shear‐induced formation of *sp* ^2^ C atoms nearby. These results confirm that plastic shear flow promotes the formation of excluded volumes around lower‐valent dopants, thereby facilitating the localization of *sp* ^2^ C atoms and ultimately leading to the formation of pore embryos with *sp* ^2^ C walls.

To elucidate the role of dopants in the nucleation of pore embryos, we introduce another pair correlation function *g*_X − p_(*r*) which quantifies the spatial distribution of “pore elements” around dopant atoms. Pore elements are identified using ghost atoms with a radius of 1.0 Å, placed at grid points with a 0.3 Å spacing in all directions (Figure 6a). We define a ghost atom as a pore element if it does not overlap with any of the real atoms (atomic radii are taken from Ref. [34]). *g*_X − p_(*r*) is calculated as:

(2) $$g_{X-p}\;\left(r\right)=\frac{1}{n_{t}}\;\sum_{t}^{n_{t}}\sum_{i}^{n_{X}}\sum_{j}^{n_{p}}\frac{V\left(t\right)}{n_{X}n_{p}\left(t\right)}\frac{\delta_{X-p}\left(r,t\right)}{4\pi r^{2}dr},$$

where *n*_p_(*t*) is the total number of pore elements at time *t* (the schematic illustration for this pore analysis is shown in Figure 6b). Figure 6c shows *g*_X − p_(*r*) for F, O, N, and Si with *C*_X_ =  4.6 at. %. Peaks in *g*_X − p_ appear right above the cutoff radii, indicating localized pore formation near dopants. In contrast, *g*_C − p_ exhibit no distinct peaks, suggesting a more homogeneous distribution of C atoms in the bulk. Interestingly, the peak intensity of *g*_X − p_ decreases with increasing valency. This indicates that mono‐ and di‐valent dopants are more effective at initiating pore embryos than tri‐ and tetra‐valent dopants, which tend to form three and four bonds with neighboring C atoms, respectively. We further differentiate between non‐aromatic (C_NA_) and aromatic C (C_A_) atoms and calculate $g_{C_{\mathrm{NA}}-p}$ and $g_{C_{A}-p}$, respectively. For all dopants, $g_{C_{A}-p}$ exhibits a broad peak at the cutoffs, followed by a gradual decay up to ∼2 Å, while $g_{C_{\mathrm{NA}}-p}$ shows no peaks. This confirms our expectation that the presence of aromatic rings is associated with the formation of pores.

Overall, the results in Figures 4, 5, 6 reveal that *sp* ^2^ C walls preferentially form around pores, particularly for low‐valent dopants, while pore stabilization is driven by the formation of aromatic pore walls. Si does not promote the formation of pores and aromatic rings. In contrast, low‐valent dopants favor the nucleation of pore embryos, forming localized voids that are eventually stabilized by aromatic walls. At high $C_{X}\left(≲15\;\mathrm{at}.\;\%\right)$, these localized pores coalesce, promoting the formation of polycyclic aromatic structures. We should point out that N plays a special role in the series of the four considered dopants, since it can participate in aromatic rings, promoting the development of extended aromatic networks.

Next, we assess the effect of dopants on the growth of pore embryos by analyzing the distribution of pore volumes *V*_pore_ for F, O, N, and Si with *C*_X_ =  4.6 at. % and compare them with pure a‐C (Figure 7). Figure 7a shows that the pore number density as a function of *V*_pore_ strongly depends on doping elements. Low‐valent dopants significantly promote the formation of larger pores (*V*_pore_ > 100 Å^3^). In contrast, pure a‐C and a‐C:Si tetravalent systems exhibit a sharp decline in the curves of Figure 7a, indicating a strong suppression of larger pores.

That dopant concentration influences the formation of large pores can be seen in Figure 7b (showing the number density of pores with $V_{\mathrm{pore}}\geq \;100\;{Å}^{3}$ as a function of *C*_X_). For low‐valent dopants, increasing *C*_X_ leads to the formation of larger pores, as evidenced by the rise in pore density. However, Si shows a negligible impact on larger pore formation, with pore densities comparable to those in pure a‐C, regardless of *C*_Si_.

The growth of pore embryos and localization of plastic shear facilitate the transformation of non‐aromatic *sp* ^2^ C walls into aromatic ones. We first show that shear is preferentially accommodated in low‐density regions, and that low‐valent dopants play a pivotal role in stabilizing aromatic rings in a‐C matrices. Figure 8 presents the spatial correlations between local shear rates ${\dot{\gamma }}_{l}$ and local number densities of C atoms $n_{l}^{C}$. The Pearson correlation coefficient *r* was evaluated as $r=\frac{\sum_{i}\left({\dot{\gamma }}_{l}\left(i\right)-{\bar{\dot{\gamma }}}_{l}\right)\left(n_{l}^{C}\left(i\right)-{\bar{n}}_{l}^{C}\right)}{\sqrt{\sum_{i}{\left({\dot{\gamma }}_{l}\left(i\right)-{\bar{\dot{\gamma }}}_{l}\right)}^{2}\sum_{i}{\left(n_{l}^{C}\left(i\right)-{\bar{n}}_{l}^{C}\right)}^{2}}}$. ${\dot{\gamma }}_{l}$ was obtained from numerical differentiation of the velocity profile along the *z*‐axis. *r*_X_ for X = F, O, N, Si range from − 0.74 to − 0.5 (with P‐values < 10^−5^), indicating significant anti‐correlations between local shear rate and atomic density. These results confirm that the growth of pore embryos and the emergence of low‐density phases promote plastic shear localization. However, the causality between these two quantities is difficult to identify because local density reduction and shear localization are strongly coupled and concurrent processes [35, 36].

Figure 9 presents the lifetime distribution τ_*a*_ of aromatic C atoms for F, O, N, and Si at *C*_X_ =  4.6 at. % and comparisons with pure a‐C. For all systems, peaks appear at τ_*a*_ =  0 ps, meaning that most aromatic rings are rapidly disrupted. However, the τ_*a*_ decay strongly depends on the doping element. The decay of the τ_*a*_ curve becomes gentler as the valency decreases. Notably, a‐C:F exhibits the longest‐living aromatic C atoms with τ_*a*_ > 100 ps. For a‐C:Si and pure a‐C, aromatic C atoms with τ_*a*_ > 30 ps are not observed. Plotting the averaged lifetimes 〈τ_*a*_〉 as a function of *C*_X_ also highlights a clear distinction between tetravalent systems (a‐C and a‐C:Si) and a‐C systems containing low‐valent elements. For Si, 〈τ_*a*_〉 remains unaffected by the increase in *C*_X_, resembling the trend seen in pure a‐C. In contrast, for low‐valent dopants, 〈τ_*a*_〉 increases with *C*_X_. This trend is particularly pronounced for N, as various N‐containing aromatic rings can form, whereas mono‐ and di‐valent atoms tend to introduce structural defects in polycyclic aromatic regions.

Taken together, the results demonstrate that dopants with valency less than four serve as key promoters of shear‐induced aromatization in a‐C, effectively creating and stabilizing larger pores and polycyclic aromatic structures. In contrast, tetravalent Si acts as an inhibitor, which collapses pore embryos and disrupts aromatic C. This distinct contrast in dopant effects highlights the decisive role of the dopant's chemical nature in dictating the structural evolution of a‐C under shear. The mechanisms provide atomistic insights into how the combination of chemical impurities and shear deformation catalyzes a swift transition from a‐C to amorphous graphene, thereby unlocking its superlubric behavior at the interface.

## 3 Discussion

The systematic and comprehensive dataset of QM MD trajectories reveals that shear‐induced aromatization in a‐C is driven by the chemo‐mechanical interplay of chemical impurities and plastic shear deformation. Even in well‐controlled laboratory experiments, a‐C surfaces are inevitably contaminated by environmental species, e.g., O_2_ and H_2_O, during deposition processes or subsequent air exposure. Moreover, upon tribological load, the surface chemical composition changes significantly via tribochemical reactions with lubricants [12, 37]. Our results suggest that, in addition to ordinary intrinsic doping during deposition, such extrinsic doping of a‐C plays a pivotal role in the emergence of polycyclic aromatic interfaces. The structure‐property maps in Figure 1 elucidate the hitherto unknown, complex relationships between doping element, concentration, friction coefficient, and friction regime. These findings provide the first in silico screening of dopants in a‐C for promoting or hindering the shear‐induced formation of superlubric amorphous graphene. Shear‐induced deformation is another key driving force that induces localized plastic flow and bond rearrangements. Accordingly, initial high‐friction CW of asperity contacts is essential for effectively releasing mechanical energy into local regions to trigger bond breaking and promote interfacial mixing. Entering the CW regime, in turn, requires a sufficiently high contact pressure [12, 35]. In this sense, the interplay between shear and contact pressure is crucial.

In general, compressive pressure can suppress pore formation, whereas tensile stress can promote it in solids. Indeed, the density of a‐C under shear deformation increases with increasing pressure [38], in line with the trend described in Figures S6 and S7. However, it was shown that for pressure values below 20 GPa, even the density of shear‐amorphized diamond approaches the density of graphite and remains below 2.5 g cm^−3^ [38], indicating the abundance of *sp* ^2^‐hybridized C atoms upon shear even under relatively high normal pressure values. In our simulations, nano‐void nucleation and growth are driven by the interplay between plastic shear deformation and chemical stabilization by low‐valent dopants, as illustrated in Figure 3. Continuous plastic shear flow promotes local atomic rearrangements and density fluctuations, leading to the formation of pore embryos, which would subsequently collapse in pure carbon. Low‐valent dopants can passivate dangling bonds, stabilize undercoordinated C atoms, and create local excluded volumes in which the graphitic carbon phase is thermodynamically stable. Therefore, during the aromatization process, pressure tends to close voids, whereas shear and dopants promote their nucleation, survival, and growth.

Dopants’ valencies and vdW radii determine each step of shear‐induced aromatization. While other atomic properties such as mass, bond strength, and electronegativity might influence this process, our results are primarily explained by these two parameters. The initial step in the process involves the nucleation of pore embryos in a‐C, as illustrated in Figure 3a. This is dictated by the passivation of C dangling bonds with mono‐ and di‐valent dopants, resulting in the formation of excluded volumes in their vicinity—precisely the origin of pore embryos. The mono‐ and di‐valent dopants passivate C dangling bonds with C─X single bonds and C═X keto‐type bonds, respectively. Trivalent N and P tend to form two or three C─X bonds in a‐C matrices, reducing their ability to nucleate pore embryos. However, their unique chemical property is their capacity to be part of aromatic rings. While mono‐ and di‐valent dopants act as perturbations in polycyclic networks by forming nano‐voids within them, trivalent N can eliminate such defects. In contrast, tetravalent Si lacks passivation modes and preferentially forms close‐packed *sp* ^3^ C domains, hindering pore growth and stabilization. C can passivate itself via aromatic rings, whose stabilization requires two opposing aromatic rings. However, in general, such a local configuration is hindered by the presence of dangling bonds.

The influence of atomic size is also evident when examining aromatization probability within the same group, as shown in Figure 2. For example, among the mono‐ and di‐valent dopants, those with larger atomic sizes exhibit a more pronounced impact on pore nucleation and growth, as evidenced by detailed analyses (Figures 4, 5, 6, 7, 8, 9). Thus, a dopant with lower valency and a larger atomic size emerges as the most potent aromatization promoter. Interestingly, N deviates from this trend due to its peculiar chemical nature, as mentioned above. Furthermore, optimal concentrations of low‐valent dopants localize the shear zone while maintaining sufficiently high shear stress in a‐C to sustain plastic flow and atomic mobility. In contrast, excess dopant concentrations reduce shear stress and atomic mobility, resulting in non‐aromatic passivation with terminating functional groups [16, 22]. High *C*_X_ increases steady‐state friction due to increased surface corrugation and instability of terminating functional groups, consistent with experimental results by Chen et al. [20], who showed the correlation of the graphitization process with surface H depletion. Our results suggest that an optimal local concentration of lower‐valent dopants is $C_{X}≲15$ at. %, rather than macroscopic experimental concentrations.

This study focuses on single‐doped systems; however, co‐doping is likely to be relevant to real‐world surfaces. It is interesting to examine, at least preliminarily, the transferability of the knowledge obtained for single‐doped systems to co‐doped systems. Our simulations of two co‐doped systems, namely a‐C:Si:H and a‐C:Si:O (Figure S8), show that two dopants can function either independently or cooperatively. In the combination of tetra‐valent Si and mono‐valent H, the two dopants behave rather independently, i.e., H promotes aromatization while Si prevents it, and aromatic and *sp* ^3^‐bonded networks coexist in the matrices. In contrast, replacing H with O leads to a different frictional response. O preferentially binds to Si, generating an oxide phase in which siloxane (Si─O─Si) bridges locally passivate Si dangling bonds. Although this siloxane passivation allows aromatic domains to form, these domains are likely to compete with the silicon oxide phase that accommodates plastic shear. If the latter dominates, the emergence of low‐friction interfaces is suppressed. The cooperative interaction between Si and O can thus be important for generating shear‐accommodating phases. However, non‐oxidized Si atoms can still act as local aromatization inhibitors, highlighting the importance of the system stoichiometry.

Importantly, our theoretical findings, namely that doping with low‐valent elements can trigger superlubricity in a‐C whereas tetravalent elements cannot, are in excellent agreement with previous experiments on ta‐C [39], a‐C:H [19], a‐C:F [40], a‐C:Cl [41], ta‐C:N [42], a‐C:S:F [43], and SiC [44] mostly conducted in high vacuum (Table S1). It is also worth noting that doping has multiple simultaneous effects as it can affect other properties of a‐C tribological interfaces, e.g., potential energy corrugation [15], mechanical properties [14], and surface morphologies. Si doping improves thermal stability [45] and adhesion onto steel [46], and is effective for shielding the moisture effect [20]. Cl doping has been shown to reduce friction [41] but may also promote corrosion. S can accelerate a‐C's severe wear [37]. Nevertheless, our results suggest that a strategic approach could mitigate such trade‐offs. For example, a possible implementation strategy could rely on chemical vapor deposition of a coating with a well‐controlled chemical composition to maintain bulk mechanical stiffness and in situ mechanochemical doping with low‐valent elements from lubricants exclusively at the topmost layer. This combined approach with intrinsic and extrinsic doping could regulate shear‐induced aromatization without sacrificing mechanical properties or chemical stability. The kinetics of this mechanochemical doping can be controlled by tailoring chemical functionalities in adsorbed molecules [12, 47] and adjusting local contact pressures [37], which are determined by bulk mechanical stiffness and local slope topography [48].

In conclusion, this study proposes a generalized theoretical concept for the mechanochemical synthesis of polycyclic amorphous graphene‐like surfaces from a‐C, driven by the chemical‐mechanical synergy between impurities and shear deformation. This strategy holds strong potential for controlling macroscale superlubricity. The systematic screening of dopants reveals that tetravalent a‐C matrices inhibit in situ aromatization, whereas lower‐valent dopants serve as effective promoters. This aromatization scenario can be extended to heavier elements (such as metals) and more complex ternary systems [12]. The self‐healing, ‐adaptive, and ‐lubricating nature of aromatic interfaces makes them promising for engineering applications where severe solid‐solid contacts are inevitable. While an initial decoration of surfaces with an aromatic layer is rapidly consumed under boundary lubrication, a self‐forming and ‐healing aromatic surface persists. Overall, these findings not only offer a data‐driven recipe for designing sustainable carbon nanostructures but also open a novel mechanochemical route for synthesizing aromatic compounds and 2D materials under tribological loads.

## 4 Methods

### 4.1 Quantum‐Mechanical Molecular Dynamics Simulations

We performed approximately 1,C000 1‐ns‐long QM MD simulations using the self‐consistent‐charge DFTB method [27], implemented in the Atomistica package [49]. We prepared a‐C bulk samples with varying *C*_X_ (≲22 at. %) of eight different dopants. For each *C*_X_, six different samples in a cubic cell of 11.25 × 11.25 × 11.25 Å^3^ were prepared. The corresponding initial density for pure a‐C was 3.0 g cm^−3^. Variations in the system density during shear are presented in Figure S9. Each sample was generated independently from a random initial atomic arrangement. After structural relaxation using the FIRE algorithm until all force components acting on the atoms were below 0.05 eV Å^−1^, the systems were melted at *T* = 5000 K for 10 ps in the NVT ensemble using a Peters stochastic thermostat and subsequently cooled down to 0 K over 50 ps. The systems were equilibrated at *P* = 5 GPa and *T* = 300 K for 10 ps before shear was applied. Thus, the six MD trajectories at a given concentration for a given dopant system are statistically independent. The dopant clustering observed is not an artifact of the initial atomic arrangement, but a thermodynamically driven feature of the modeled a‐C systems. A constant shear velocity *v* of 100 m s^−1^ was imposed using Lees‐Edwards boundary conditions (BCs) [28], and a constant pressure *P* of 5 GPa in all three directions was imposed with a Berendsen barostat [50]. The applied pressure of 5 GPa is realistic for hard coatings such as a‐C, since surface asperities can experience such high local contact pressures under tribological loads [37]. Simulations at different pressures show that the probability of observing complete interface passivation by aromatization decreases as pressure increases above 1 GPa (Figures S6 and S7). Within the computationally feasible range, the effect of sliding velocity on the aromatization process is not critical, provided that the accumulated shear strain is the same (Figure S10). The system temperature *T* was kept at 300 K using a Peters thermostat [51] with a cutoff of 5 Å. The equations of motion were integrated with a time step of 0.5 fs using the velocity Verlet algorithm [52]. The shear stress *τ* shown in Figure 1 was calculated from the *σ*_zx_ component of the stress tensor and averaged over the last 0.1 ns. The statistical robustness of our dataset is supported by the sufficiently small standard deviations in Figures 4, 5, 6, 7, 8, 9 and by an expanded a‐C:O dataset from 90 to 150 trajectories (Figure S11), which yields almost the same probability of aromatization (0.167 vs. 0.173). For interatomic interactions, long‐range electrostatic Coulomb interactions were included within the DFTB framework. DFTB has been shown to accurately model aromatic tribological interfaces in reasonable agreement with experiments [6, 12, 16]. The accuracy and validity of the DFTB MD results were verified by first‐principles DFT MD simulations (Figure S1). In particular, shear‐induced aromatization and passivation were observed during 0.4‐ns‐long DFT MD. For these auxiliary simulations, we employed the mixed Gaussian and Plane Wave method [53] and the Perdew‐Burke‐Enzerhof exchange correlation functional [54], as implemented in the CP2K software package. Gaussian double‐zeta basis sets with polarization functions were used for the valence electrons, and Goedecker‐Teter‐Hutter pseudopotentials [55] were applied to take into account the effects of the core electrons. Further details of the DFT calculations are provided in Supporting Information. Additionally, we confirmed that the choice of BCs and barostat does not affect the simulation results or conclusions by conducting supplementary QM MD simulations with different setups, including conventional two‐slab sliding simulations [12] and Lees‐Edwards BCs with fixed lateral dimensions (Figure S12). Regarding the selection of dopants, it is worth noting that the periodic table of dopants shown in Figure 1 does not include all common dopants (e.g., B [56] and Al are missing), due to the unavailability of reliable DFTB parametrizations for these elements. To partially address this limitation, we performed a DFT MD simulation of a‐C:B with *C*_B_ =  4.6 at. %. The results are shown in Figure S1 and clearly show the onset of shear‐induced aromatization. Visualization of simulation atomic configurations was carried out with OVITO [57].

### 4.2 Trajectory Analyses

To differentiate between the high‐friction CW and low‐friction passivation regime, we replicated the system twice along the *z*‐axis and checked whether the a‐C system consists of a single block *n_b_* (*t*) =  1 or two disconnected blocks *n_b_* (*t*) =  2 at each 0.2 ps interval over the last 0.1 ns. The CW index *I*_cw_(*t*) at time *t* was defined as:

(3) $$I_{\mathrm{cw}}\;\left(t\right)=\left\{\begin{array}{c} 1\;\mathrm{if}\;n_{b}\left(t\right)=1\;\\ 0\;\mathrm{if}\;n_{b}\left(t\right)=2\; \end{array}\right.\;$$

When the averaged CW index 〈*I*_cw_(*t*)〉 exceeds 0.2, we considered the trajectory as a CW system. For a non‐CW trajectory, we further classified it into the non‐aromatic and aromatic passivation regimes, based on surface aromaticity analysis. First, we subdivided the system into grids of 2.5 × 2.5 Å^2^ along the *x* and *y* directions to account for surface roughness. Atoms within 2.5 Å of the highest and lowest atoms on the lower and upper surfaces, respectively, were defined as surface atoms. For all surface atoms, the degree of aromaticity $D_{a}^{i}\left(t\right)$ of atom *i* at time *t* was calculated by using the shortest‐path ring statistics algorithm [58] and the harmonic oscillator model of aromaticity for heterocycle electron delocalization [30]. Only 5‐ and 6‐membered rings were considered in the analysis. The aromaticity index $I_{a}^{i}\left(t\right)$ of atom *i* was defined as:

(4) $$I_{a}^{i}\;\left(t\right)=\left\{\begin{array}{c} 1\;\mathrm{if}\;D_{a}^{i}\left(t\right)\geq 0.5\;\\ 0\;\mathrm{otherwise}\; \end{array}\right.\;$$

The lifetime of aromatic C atoms was defined as the time period during which $I_{a}^{i}\;\left(t\right)=1$. The surface aromaticity index ${\bar{I}}_{a}$ was calculated by averaging the atomic aromaticity indices over all surface C atoms and time‐averaging over the last 0.1 ns. A system was classified as exhibiting shear‐induced aromatization if the surface aromaticity index ${\bar{I}}_{a}\geq 0.5$ for at least one of the two surfaces. This classification protocol is also found in Ref. [29].

We performed pore analysis using ghost atoms to detect nano‐voids in a‐C matrices. We subdivided the simulation cell into grids of 0.3 × 0.3 × 0.3 Å^3^ and placed a ghost atom with a radius *R*_g_ of 1 Å at each grid point. The radii of C and dopant atoms were defined as their covalent radii *R*_X_  [34]. A ghost atom was considered a pore element if the distance between a real atom and the ghost atom exceeded the cutoff *R*_cut_(  = *R*_g_  + *R*_X_). The pore volume was then estimated by placing cubic boxes with a size of 0.3 × 0.3 × 0.3 Å^3^ at each grid point and summing the volumes of all boxes whose centers are inside the pore elements.

## Author Contributions

T.K., G.M., and M.M. conceived and designed the study. T.K., K.H., and L.M. performed the simulations and data analyses. T.K., G.M., and M.M discussed, interpreted the results, and wrote the manuscript. All authors approved the final version of the manuscript.

## Funding

This work was funded by Japan Science and Technology Agency (JST) JPMJPR22A6 (T.K.), JPMJCR2191 (T.K.), Japan Society for the Promotion of Science (JSPS) 25K01146 (T.K.). Fraunhofer‐Gesellschaft 840066 (G.M. and M.M.). European Research Council 101201061 (G.M. and M.M.).

## Conflicts of Interest

The authors declare no conflicts of interest.

## Supporting information




**Supporting File**: advs75566‐sup‐0001‐SuppMat.docx.

## Data Availability

The data that support the findings of this study are available from the corresponding author upon reasonable request.
