# Supplementary material for: Shear‐Induced Emergence of Aromatic Superlow‐Friction Interfaces in Amorphous Carbon: Triggering Chemical Impurities and Atomic‐Scale Mechanisms
Source: Adv Sci (Weinh). 2026 May 25;13(43):e75566. doi: 10.1002/advs.75566 (PMC13336024; doi:10.1002/advs.75566)
Supplement: Supplementary file 1 — Supporting File: advs75566‐sup‐0001‐SuppMat.docx. [file ADVS-13-e75566-s001.docx]

Supplementary Information

**Shear-Induced Emergence of Aromatic Superlow-Friction Interfaces in Amorphous Carbon: Triggering Chemical Impurities and Atomic-Scale Mechanisms**

Takuya Kuwahara^1*^, Koki Horiguchi^1^, Leonhard Mayrhofer^2^, Gianpietro Moras^2^, Michael Moseler^2,3*^

^1^Osaka Metropolitan University, Department of Mechanical Engineering, 1-1 Gakuen-cho, Naka-ku, 599-8531 Sakai, Japan

^2^Fraunhofer Institute for Mechanics of Materials IWM, MicroTribology Center μTC, Wöhlerstraße 11, 79108 Freiburg, Germany

^3^University of Freiburg, Institute of Physics, Hermann-Herder-Straße 3, 79104 Freiburg, Germany

^*^Corresponding authors: [kuwa@omu.ac.jp](mailto:kuwa@omu.ac.jp); [michael.moseler@iwm.fraunhofer.de](mailto:michael.moseler@iwm.fraunhofer.de)

We performed ab-initio DFT MD simulations of a-C, a-C:O, and a-C:B using the CP2K code^[1]^, where the mixed Gaussian and Plane Wave method^[2]^ was employed. A plane-wave cutoff energy of 500 Ry and relative cutoff of 40 Ry with 5 multigrids were chosen to define the grid spacing. The convergence criterion of the self-consistent field loop was set to $1\times{10}^{-5}$ Ha. All calculations were performed within the Perdew-Burke-Enzerhof approximation^[3]^ to the exact exchange correlation functional. Gaussian double-zeta basis sets with polarization functions were used to expand the Kohn-Sham wave functions of the valence electron and Goedecker-Teter-Hutter pseudopotentials^[4]^ were applied to effectively treat the core electrons. Periodic boundary conditions were imposed along all three directions and the Brillouin zone was sampled at the Γ point. In the MD simulations, after pressure equilibration at an external pressure of 5 GPa, a shear velocity of 100 m s^-1^ is imposed with Lees-Edwards boundary conditions^[5]^. The DFT MD sliding simulations are carried out for 400 ps. The system temperature *T* was kept constant at 300 K using a Langevin thermostat^[6]^ with a time constant $\tau_{T}$ of 100 fs, and the equations of motion are integrated with a time step ∆*t*  of 2.0 fs using the velocity-Verlet algorithm. The normal pressure of 10 GPa is controlled using a Berendsen barostat^[7]^ with a time constant $\tau_{P}$ of 100 fs.

| 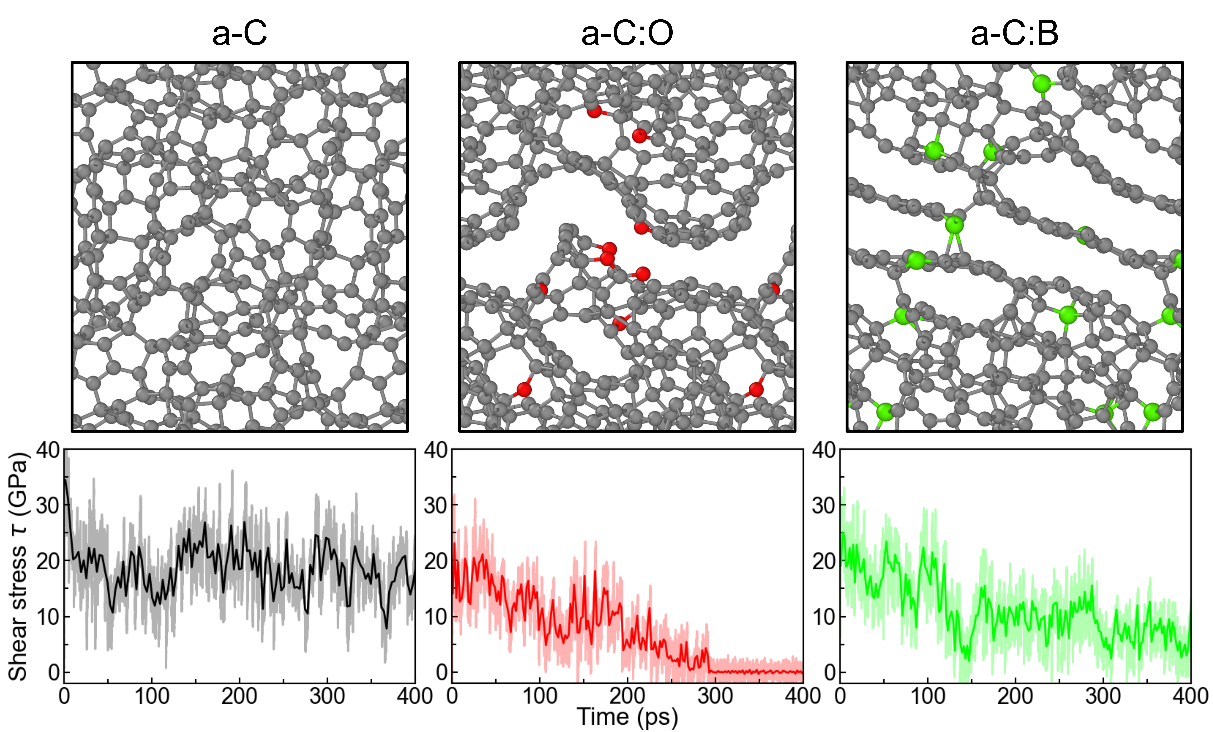 |
| --- |
| **Figure S1.** DFT MD simulations of a-C (left), a-C:O (middle), and a-C:B (right) with the Lees-Edwards boundary conditions. A dopant concentraion of 4.6 at. % was employed. |

| 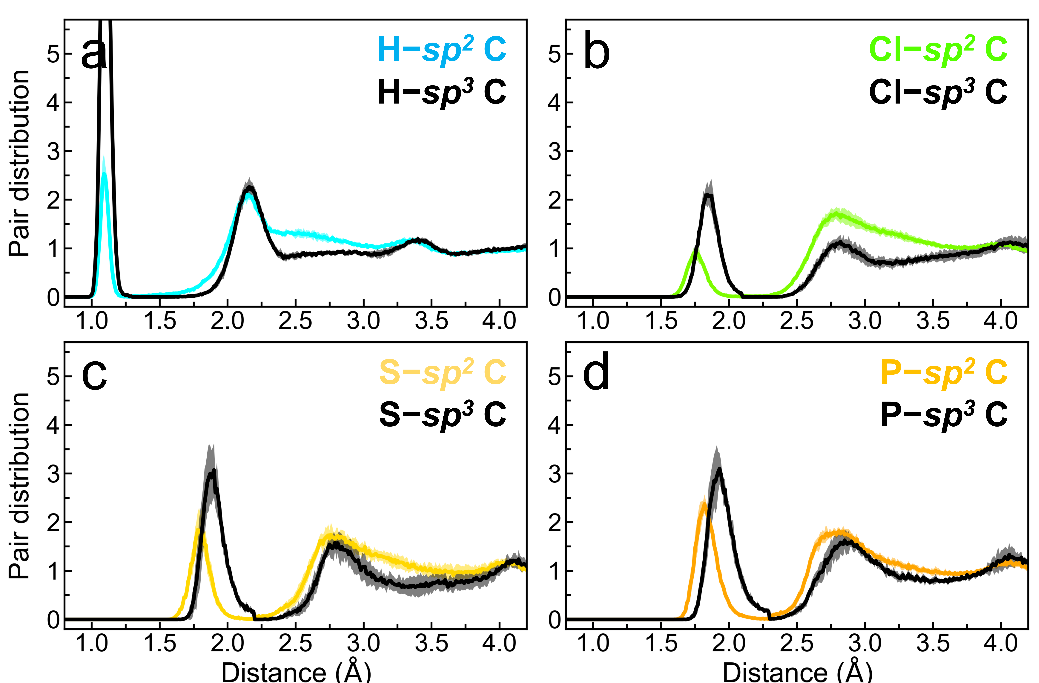 |
| --- |
| **Figure S2.** Pair correlation functions between dopants and *sp*^2^ carbon $g_{X-{sp}^{2} C}$ and between dopants and *sp*^3^ carbon $g_{X-{sp}^{3} C}$: (a) a-C:H, (b) a-C:Cl, (c) a-C:S, and (d) a-C:P at a dopant concentraion of 4.6 at. %. These pair correlation functions are calculated every 0.2 ps and averaged between 200 and 450 ps with a bin width is 0.01 $Å$. Shaded regions represent standard deviations of six independent MD trajectories. |

| 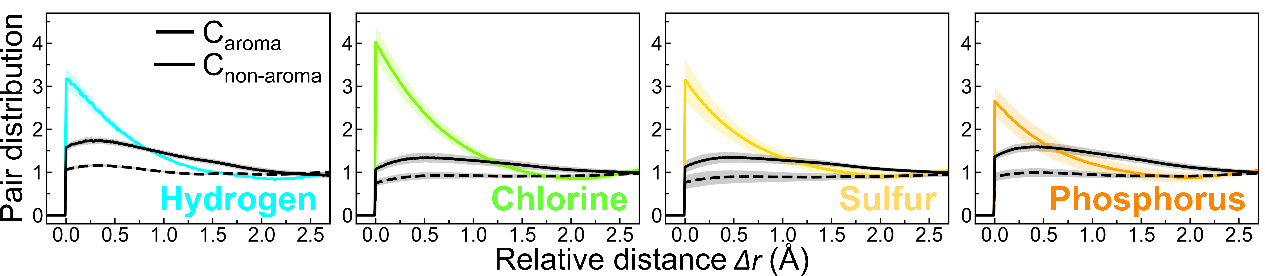 |
| --- |
| **Figure S3.** Pore statistics for a-C:H, a-C:Cl, a-C:S, and a-C:P with a dopant concentration of 4.6 at. %. Averaged pair correlation functions of dopant-pore $g_{X-p}$ (colored), aromatic carbon-pore $g_{C_{A}-p}$ (solid black), and non-aromatic-carbon-pore $g_{C_{\mathrm{NA}}-p}$ (dashed black). This analysis is carried out every 0.2 ps between 200 and 450 ps. Shaded regions represent standard deviations of six independent MD trajectories. Aromaticity indices $I_{a}(t)$ of carbon atoms at a given time *t* are evaluated using a geometrical measure of aromaticity. When a carbon atom, which is a part of 5- or 6-membered rings, has $I_{a}(t)\geq0.5$, it can be seen as an aromatic atom. |

| 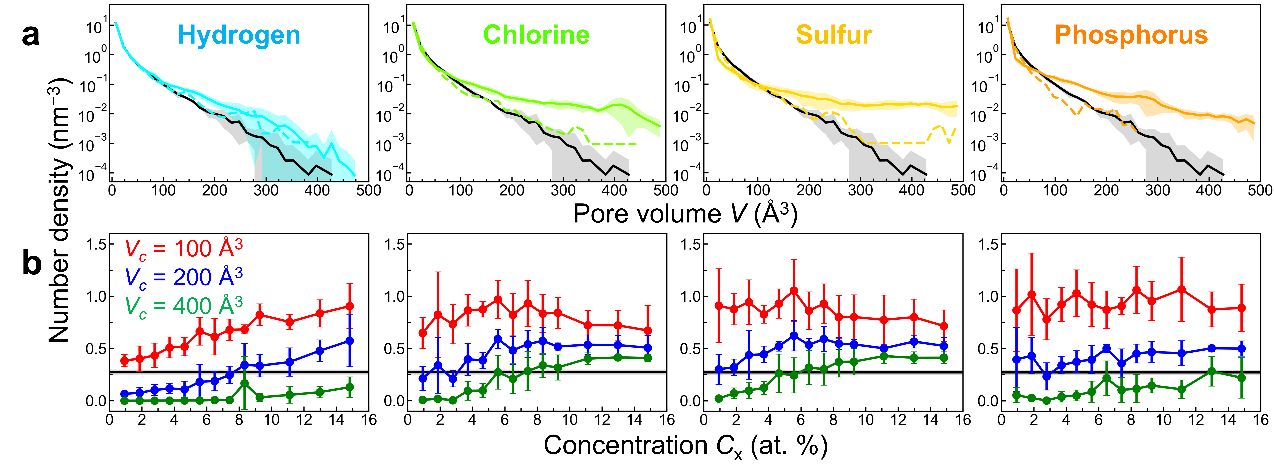 |
| --- |
| **Figure S4.** (a) Number densities (nm^−3^) of pores as a function of their volumes for a-C:H, a-C:Cl, a-C:S, and a-C:P with a dopant concentration of 4.6 at. %. The analyses are carried out between 200 and 450 ps every 0.2 ps. Solid lines and shaded regions represent averages and standard deviations of six independent MD trajectories, respectively. The results for a-C are shown by the black lines. (b) Number densities of pores with volumes larger than a threshold volume $V_{c}$ as a function of the dopant concentrations: $V_{c}=100$ (red), 200 (blue), and 400 $Å^{3}$ (green). The marker plots and error bars represent averages and standard deviations of six independent MD trajectories, respectively. The black lines and shaded regions represent represent averages and standard deviations of six independent MD trajectories, respectively, for pure a-C with $V_{c}=100 Å^{3}$ . |

| 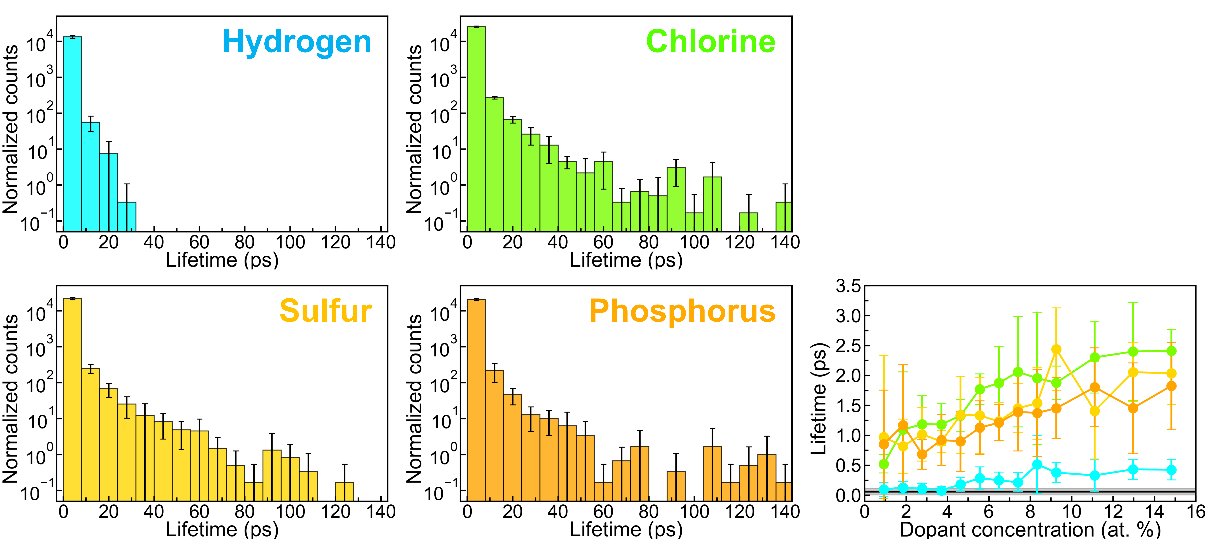 |
| --- |
| **Figure S5.** Lifetime distributions of aromatic carbon for a-C:H, a-C:Cl, a-C:S, and a-C:P with a dopant concentration of 4.6 at. % and averaged lifetimes of aromatic carbon as functions of the dopant concentrations. The analyses are carried out between 200 and 450 ps every 0.2 ps. For each bin, the counts are normalized to the total number of carbon atoms. The bar plots and error bars represent averages and standard deviations of 6 independent MD trajectories for each system. Black horizontal lines and shaded gray regions also represent averages and standard deviations of 6 independent MD trajectories for pure a-C, respectively. |

| *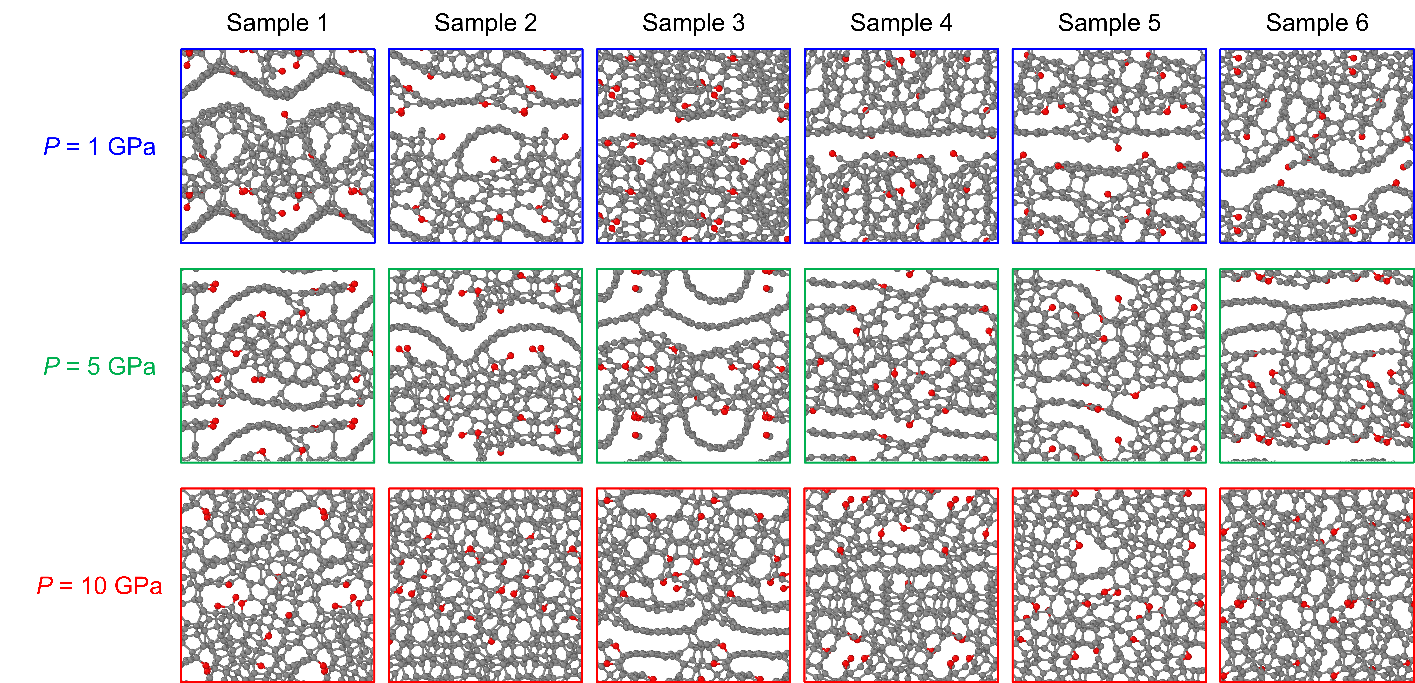* |
| --- |
| **Figure S6.** DFTB MD simulations of a-C:O with 4.6 at. % O at different pressures (*P* = 1, 5, and 10 GPa) using the Lees-Edwards boundary conditions. Six independent 1-ns-long MD trajectrories were generated for each pressure. |

| *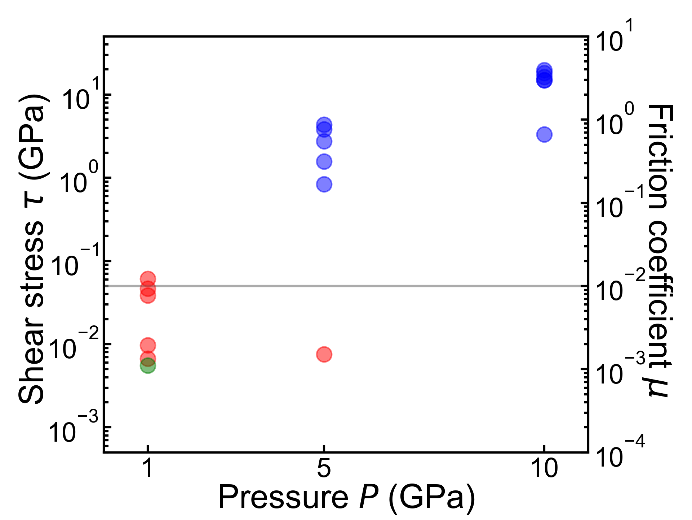* |
| --- |
| **Figure S7.** Structure-property map of a-C:O with 4.6 at. % O at different pressures. The probabilities for full aromatic interface passivation are 0.83, 0.17, and 0.00 for *P* = 1, 5, and 10 GPa, respectively. |

| 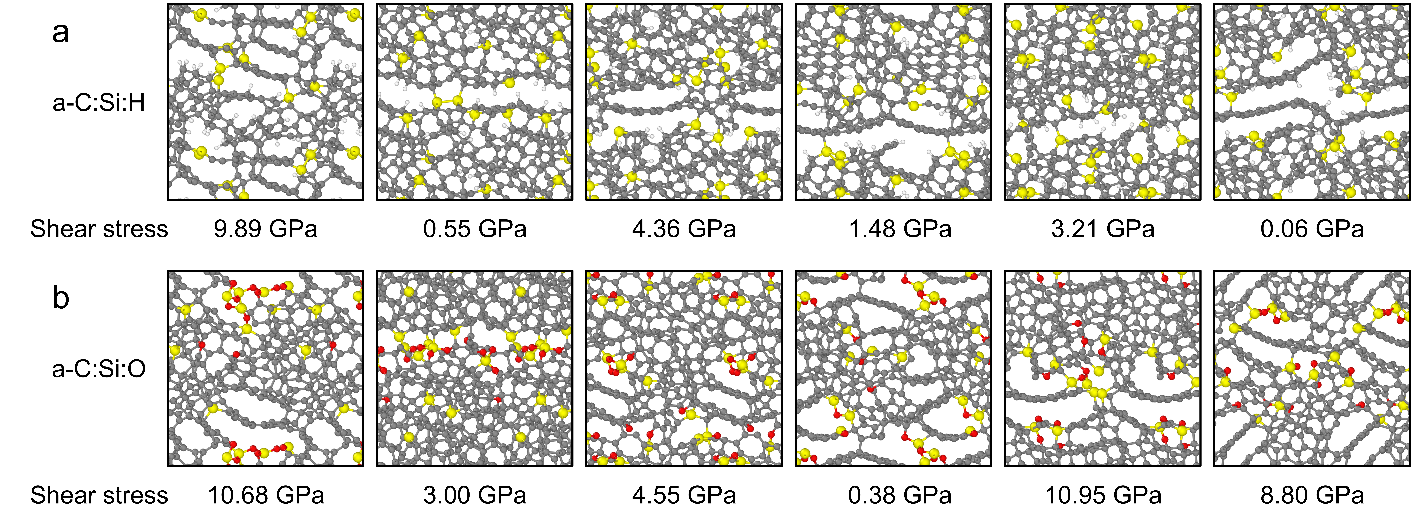 |
| --- |
| **Figure S8.** DFTB MD simulations of (a) a-C:Si:H and (b) a-C:Si:O using the Lees-Edwards boundary conditions. In a-C:Si:H, a silicon concentraion of 4.6 at. % and a hydrogen concentration of 9.3 at. % were employed. In a-C:Si:O, a silicon concentraion of 4.6 at. % and an oxygen concentration of 4.6 at. % were employed. |

**Table S1.** A summary of previous experiments on a-C:X.

| Materials | Dopant concentrations *C*_X_ (at. %) | Environments | Friction coefficients | References |
| --- | --- | --- | --- | --- |
| a-C:H | 39.3 | Dry N_2_ | 0.001 | ^[8]^ |
| a-C:F | 7.4 | High vacuum  ($2\times{10}^{-4}$ Pa) | 0.02 | ^[9]^ |
| ta-C:N | ~10 | High vacuum  ($5\times{10}^{-4}$ Pa) | 0.013 | ^[10]^ |
| a-C:Cl | 13.7 | unlubricated | 0.05 | ^[11]^ |
| a-C:F:H | 2.1 (F)  10.3 (H) | In air, low humidity (RH 15%) | 0.010 | ^[12]^ |
| a-C:O:H | 5.4 (O)  37.4 (H) | Dry N_2_ | 0.004 | ^[13]^ |
| a-C:S:F | 8.0 (S)  11.5 (F) | High vacuum  ($<5\times{10}^{-4}$ Pa) | 0.01−0.02 | ^[14]^ |
| ta-C |  | High vacuum  ($<1\times{10}^{-3}$ Pa) | 0.50 | ^[15]^ |
| a-C:Si:H | 9.5 (Si)  15 (H) | High vacuum  ($8\times{10}^{-3}$ Pa) | 0.13 | ^[16]^ |
| a-C:Si:H | - (Si)  12 (H) | High vacuum  ($1\times{10}^{-3}$ Pa) | 0.55 | ^[17]^ |
| Polycrystalline SiC | ~50 | High vacuum  ($7\times{10}^{-3}$ Pa) | 0.6−0.7 | ^[18]^ |

| 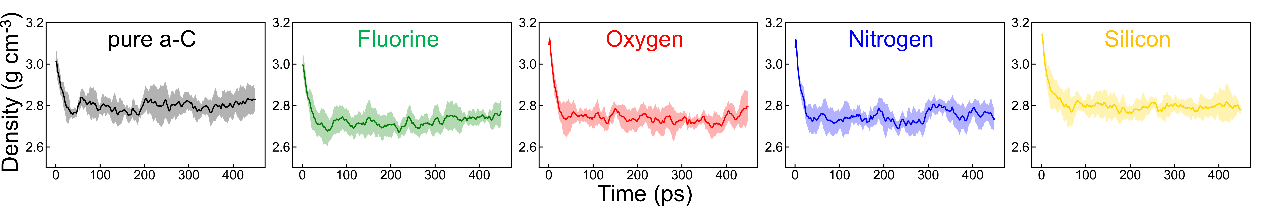 |
| --- |
| **Figure S9.** Variations in the density under shear for pure a-C, a-C:F, a-C:O, a-C:N, and a-C:Si with dopant concentration of 4.6 at. %. For each plot, solid lines and semi-transparent regions represent the averages and standard deviations over 6 independent MD trajectrories, respectively. The results indicate that the density decreases imediately and reach a steady-state value, which is not affected by differences in the initial density. |

| 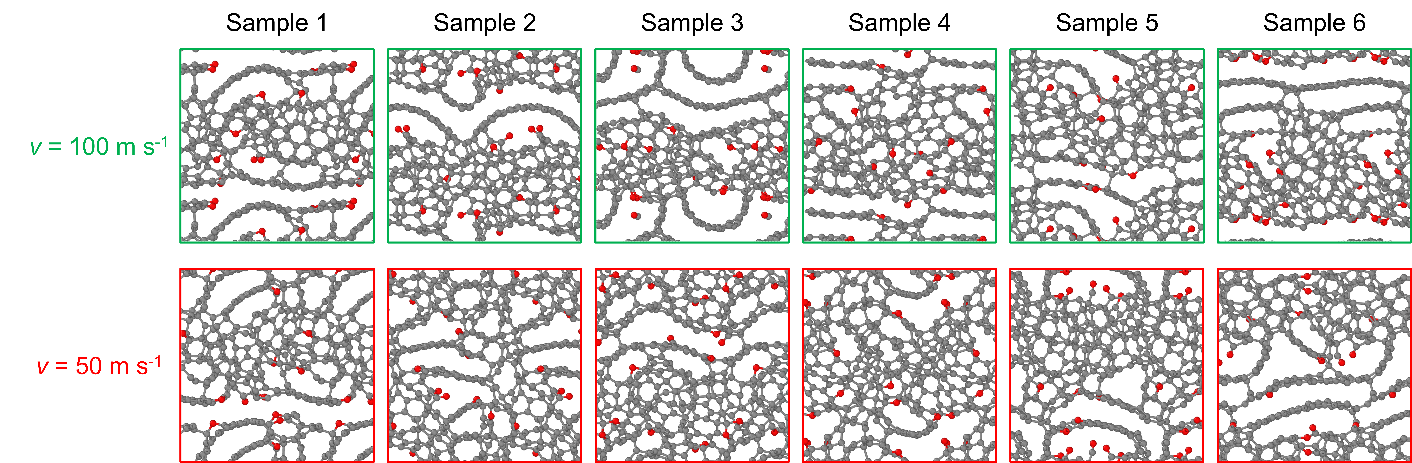 |
| --- |
| **Figure S10.** DFTB MD simulations of a-C:O with 4.6 at. % O at three different shear velocities using the Lees-Edwards boundary conditions. Six independent 2.0-ns-long MD trajectories were generated for the lower velocity (50 m s^-1^) to ensure the same total sliding distance (100 nm). |

| *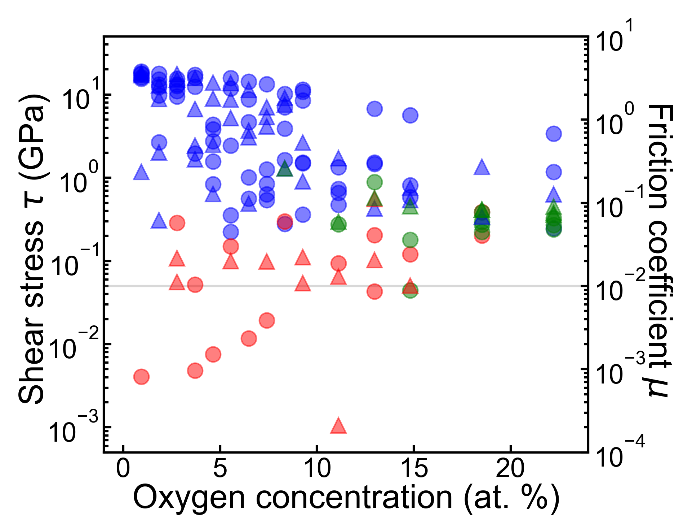* |
| --- |
| **Figure S11.** Structure-property map of a-C:O obtained from 150 independent MD trajectories (10 for each oxygen concentration). 90 trajectories are from the original dataset shown in Fig. 1a (circle) and the rest was newly generated (triangle). |

| 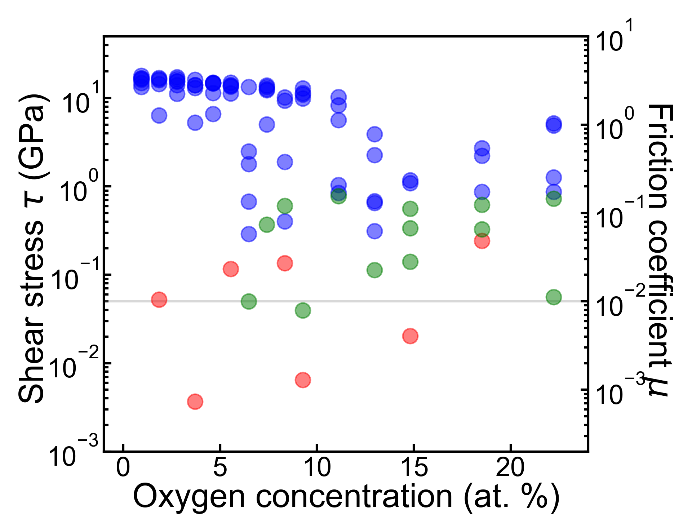 |
| --- |
| **Figure S12.** Shear stress and friction regime mapping with varying oxygen concentrations. These MD simulations are peformed with the same Lees-Edwards boundary condtions as in Fig. 2 but the Brendsen barostat is coupled only to the z direction of the simulation cell to keep the lateral dimension unchaged. The results indicate that the shear-induced aromatization observed in this study is not affected by the setup of the barostat, i.e. changes in the cell shape. Each marker represents the result of a MD trajectory and is colored according to the structural analysis described in Ref. ^[19]^: cold-welding (blue), aromatic passivation (red), and non-aromatic passivation (green). For each oxygen concetration, 6 MD trajectories are generated with different initial configurations. |

**References**

[1] T. D. Kühne, M. Iannuzzi, M. Del Ben, V. V. Rybkin, P. Seewald, F. Stein, T. Laino, R. Z. Khaliullin, O. Schütt, F. Schiffmann, D. Golze, J. Wilhelm, S. Chulkov, M. H. Bani-Hashemian, V. Weber, U. Borštnik, M. Taillefumier, A. S. Jakobovits, A. Lazzaro, H. Pabst, T. Müller, R. Schade, M. Guidon, S. Andermatt, N. Holmberg, G. K. Schenter, A. Hehn, A. Bussy, F. Belleflamme, G. Tabacchi, A. Glöß, M. Lass, I. Bethune, C. J. Mundy, C. Plessl, M. Watkins, J. VandeVondele, M. Krack, J. Hutter, *J. Chem. Phys.* **2020**, *152*, 194103.

[2] G. Lippert, J. Hutter, M. Parrinello, *Mol. Phys.* **1997**, *92*, 477.

[3] J. P. Perdew, K. Burke, M. Ernzerhof, *Phys. Rev. Lett.* **1996**, *77*, 3865.

[4] S. Goedecker, M. Teter, J. Hutter, *Phys. Rev. B* **1996**, *54*, 1703.

[5] A. W. Lees, S. F. Edwards, *J. Phys. C Solid State Phys.* **1972**, *5*, 1921.

[6] D. Frenkel, B. Smit, *Understanding Molecular Simulation: From Algorithms to Applications*, 2nd Edition., Academic Press, San Diego **2002**.

[7] H. J. C. Berendsen, J. P. M. Postma, W. F. van Gunsteren, A. DiNola, J. R. Haak, *J. Chem. Phys.* **1984**, *81*, 3684.

[8] X. Chen, C. Zhang, T. Kato, X. Yang, S. Wu, R. Wang, M. Nosaka, J. Luo, *Nat. Commun.* **2017**, *8*, 1675.

[9] R. Zhang, M. Shen, Z. He, *Surf. Interface Anal.* **2020**, *52*, 339.

[10] T. Tokoroyama, C. Fujiwara, M. Murashima, M. Yamaguchi, N. Umehara, *Diam. Relat. Mater.* **2024**, *143*, 110853.

[11] Y. Tokuta, T. Itoh, T. Shiozaki, M. Kawaguchi, S. Sasaki, *Tribol. Int.* **2017**, *113*, 377.

[12] J. Cui, L. Qiang, B. Zhang, T. Yang, J. Zhang, *Surf. Interface Anal.* **2013**, *45*, 1329.

[13] X. Chen, X. Yin, W. Qi, C. Zhang, J. Choi, S. Wu, R. Wang, J. Luo, *Sci. Adv.* **2020**, *6*, eaay1272.

[14] F. Wang, L. Wang, Q. Xue, *Carbon* **2016**, *96*, 411.

[15] C. Meunier, P. Alers, L. Marot, J. Stauffer, N. Randall, S. Mikhailov, *Surf. Coat. Technol.* **2005**, *200*, 1976.

[16] S. H. Yang, H. Kong, K.-R. Lee, S. Park, D. E. Kim, *Wear* **2002**, *252*, 70.

[17] L. Wang, R. Zhang, U. Jansson, N. Nedfors, *Sci. Rep.* **2015**, *5*, 11119.

[18] K.-H. Zum Gahr, R. Blattner, D.-H. Hwang, K. Pöhlmann, *Wear* **2001**, *250*, 299.

[19] T. Kuwahara, Y. Long, M.-I. De Barros Bouchet, J. M. Martin, G. Moras, M. Moseler, *Coatings* **2021**, *11*, 1069.
